# Dose–response effects on HbA_1c_ and bodyweight reduction of survodutide, a dual glucagon/GLP-1 receptor agonist, compared with placebo and open-label semaglutide in people with type 2 diabetes: a randomised clinical trial

**DOI:** 10.1007/s00125-023-06053-9

**Published:** 2023-12-14

**Authors:** Matthias Blüher, Julio Rosenstock, Josef Hoefler, Raymond Manuel, Anita M. Hennige

**Affiliations:** 1 Helmholtz Institute for Metabolic, Obesity and Vascular Research (HI-MAG) of the Helmholtz Zentrum München, University of Leipzig and University Hospital Leipzig, Leipzig, Germany; 2 Velocity Clinical Research, Medical City, Dallas, TX USA; 3 Staburo GmbH, Munich, Germany, on behalf of Boehringer Ingelheim Pharma GmbH & Co. KG, Biberach an der Riß, Germany; 4 Boehringer Ingelheim Pharmaceuticals, Inc., Ridgefield, CT USA; 5 Boehringer Ingelheim International GmbH, Biberach an der Riß, Germany

**Keywords:** Bodyweight loss, Dual incretin agonist, Glucagon, Glucagon-like peptide-1, Obesity, Pharmacotherapy, Semaglutide, Survodutide, Type 2 diabetes

## Abstract

**Aims/hypothesis:**

The aim of this study was to assess the dose–response effects of the subcutaneous glucagon receptor/glucagon-like peptide-1 receptor dual agonist survodutide (BI 456906) on HbA_1c_ levels and bodyweight reduction.

**Methods:**

This Phase II, multicentre, randomised, double-blind, parallel-group, placebo-controlled study, conducted in clinical research centres, assessed survodutide in participants aged 18–75 years with type 2 diabetes, an HbA_1c_ level of 53–86 mmol/mol (7.0–10.0%) and a BMI of 25–50 kg/m^2^ on a background of metformin therapy. Participants were randomised via interactive response technology to receive survodutide (up to 0.3, 0.9, 1.8 or 2.7 mg once weekly [qw; dose group (DG) 1–4, respectively] or 1.2 or 1.8 mg twice weekly [DG 5 and 6, respectively]), placebo or semaglutide (up to 1.0 mg qw). Participants and all those involved in the trial conduct/analysis were blinded; the semaglutide arm was open-label. The primary endpoint was absolute change from baseline in HbA_1c_ after 16 weeks’ treatment. The key secondary endpoint was relative change from baseline in bodyweight after 16 weeks’ treatment.

**Results:**

A total of 413 participants were randomised (DG1, *n*=50; DG2, *n*=50; DG3, *n*=52; DG4, *n*=50; DG5, *n*=51; DG6, *n*=50; semaglutide, *n*=50; placebo, *n*=60). The full analysis set comprised 411 treated participants (DG6, *n*=49; placebo, *n*=59). Adjusted mean (95% CI) HbA_1c_ decreased from baseline (mean ± SD 64.7±9.2 mmol/mol [8.07±0.84%] after 16 weeks’ treatment: DG1 (*n*=41), −9.92 mmol/mol (−12.27, −7.56; −0.91% [−1.12, −0.69]); DG2 (*n*=46), −15.95 mmol/mol (−18.27, −13.63; −1.46% [−1.67, −1.25]); DG3 (*n*=36), −18.72 mmol/mol (−21.15, −16.29; −1.71% [−1.94, −1.49]); DG4 (*n*=33), −17.01 mmol/mol (−19.59, −14.43; −1.56% [−1.79, −1.32]); DG5 (*n*=44), −17.84 mmol/mol (−20.18, −15.51; −1.63% [−1.85, −1.42]); DG6 (*n*=36), −18.38 mmol/mol (−20.90, −15.87; −1.68% [−1.91, −1.45]). The mean reduction in HbA_1c_ was similar with low-dose survodutide (DG2: −15.95 mmol/mol [−1.46%]; *n*=46) and semaglutide (−16.07 mmol/mol [−1.47%]; *n*=45). Mean (95% CI) bodyweight decreased dose-dependently up to −8.7% (−10.1, −7.3; DG6, *n*=37); survodutide ≥1.8 mg qw produced greater bodyweight reductions than semaglutide (−5.3% [−6.6, −4.1]; *n*=45). Adverse events (AEs) were reported for 77.8% of survodutide-treated participants (mainly gastrointestinal), 52.5% receiving placebo and 52.0% receiving semaglutide.

**Conclusions/interpretation:**

Survodutide reduced HbA_1c_ levels and bodyweight after 16 weeks’ treatment in participants with type 2 diabetes. Dose-related gastrointestinal AEs could be mitigated with slower dose escalations.

**Trial registration:**

ClinicalTrials.gov NCT04153929 and EudraCT 2019-002390-60.

**Funding:**

Boehringer Ingelheim Pharma GmbH & Co. KG, Ingelheim, Germany.

**Graphical Abstract:**

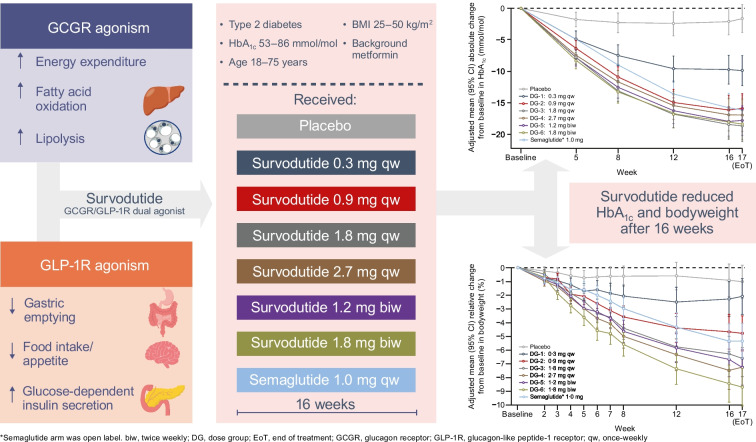

**Supplementary Information:**

The online version contains peer-reviewed but unedited supplementary material available at 10.1007/s00125-023-06053-9.


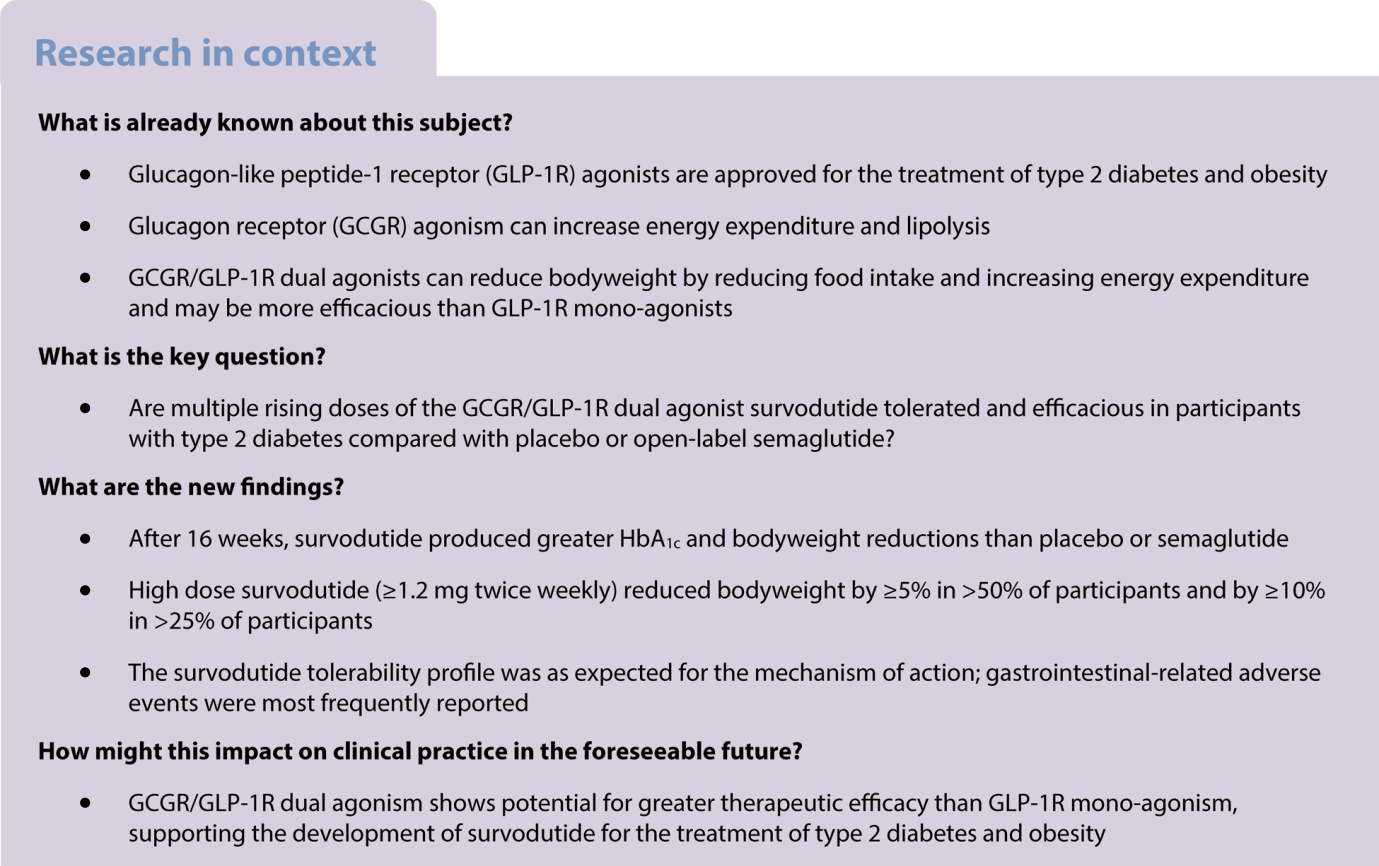


## Introduction

Glucagon-like peptide-1 receptor (GLP-1R) agonists, such as liraglutide and semaglutide, have been developed for the treatment of both type 2 diabetes and obesity. These therapies have produced placebo-corrected bodyweight decreases of up to 5.4% (liraglutide 3 mg) [1] and 12.4% (semaglutide 2.4 mg), and HbA_1c_ reductions of –12.0 to –17.5 mmol/mol (–1.1 to –1.6%) (liraglutide 1.8 mg and semaglutide 1 mg, respectively) in adults with type 2 diabetes [2, 3]. Apart from the well-characterised gastrointestinal (GI) adverse events (AEs), GLP-1R agonists are generally well tolerated [2–4]. However, dual agonists, such as glucose-dependent insulinotropic polypeptide receptor (GIPR)/GLP-1R and glucagon receptor (GCGR)/GLP-1R dual agonists, have the potential for enhanced therapeutic efficacy and improved tolerability compared with GLP-1R mono-agonists, owing to their multiple mechanisms of action [5, 6].

In addition to the glucose-lowering effects associated with GLP-1R agonism, GCGR agonism, via receptors in the liver, may lead to increased energy expenditure [7, 8]. This effect can be seen at doses that do not activate the sympathetic nervous system, thereby avoiding potentially harmful effects on the cardiovascular system [7]. GCGR signalling also leads to stimulation of hepatic glucose production (via glycogenolysis and gluconeogenesis), stimulation of lipolysis and amino acid breakdown, and suppression of hepatic fat accumulation [9].

The efficacy of GCGR/GLP-1R dual agonism has been demonstrated by oxyntomodulin, an endogenous proglucagon derivative [10]. Oxyntomodulin has been shown to reduce bodyweight and food intake in rodents and humans [11, 12] and to increase energy expenditure in people with obesity [13], via activity at both receptors. However, oxyntomodulin requires frequent dosing owing to its very short half-life; therefore, research into longer acting GCGR/GLP-1R dual agonists is warranted.

Survodutide (BI 456906) is a novel subcutaneous GCGR/GLP-1R dual agonist in development for the treatment of people with type 2 diabetes, obesity and non-alcoholic steatohepatitis (NASH). Addition of a C18 fatty acid into the acylated peptide, as a half-life-extending principle, allows for weekly administration of survodutide [14]. Preclinical studies of survodutide in murine models showed that survodutide simultaneously engages the GLP-1R and GCGR to produce reductions in bodyweight, gastric emptying and energy intake, increasing energy expenditure and improving glucose tolerance [14]. In Phase I studies (ClinicalTrials.gov NCT03175211, NCT03591718), survodutide was generally well tolerated and showed no unexpected safety or tolerability concerns in healthy volunteers and people with overweight/obesity; multiple ascending doses of survodutide over 16 weeks produced mean bodyweight decreases of up to 14.1% (2.4 mg survodutide twice weekly [biw] vs −0.3% with placebo) [15].

Here we report the results of a Phase II study (ClinicalTrials.gov NCT04153929) assessing the effects on HbA_1c_ levels and bodyweight of multiple rising doses of survodutide compared with placebo and open-label weekly semaglutide in participants with type 2 diabetes. The safety and tolerability of survodutide were also assessed. As a proof-of-clinical concept study, this trial aimed to demonstrate that survodutide lowers HbA_1c_ levels and bodyweight and to examine the dose–response relationship in this participant population to inform the design of further studies.

## Methods

### Study design and participants

**Fig. 1:**
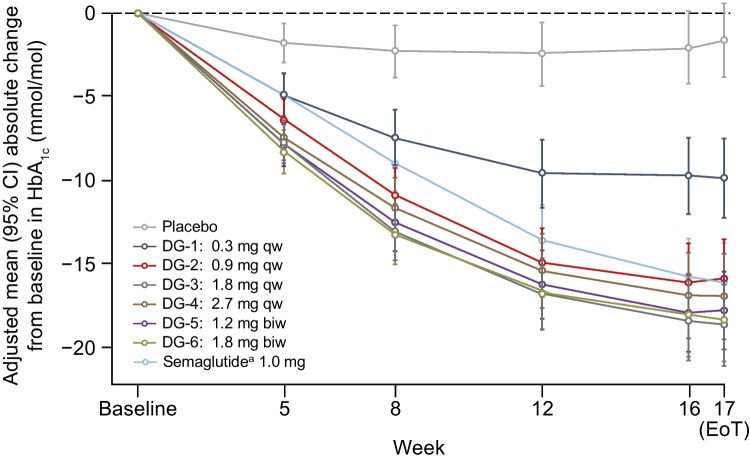
MMRM estimates for the absolute change in HbA_1c_ from baseline to EoT. ^a^Semaglutide arm was open label

This study had a multicentre, randomised, double-blind within dose groups (DGs), parallel-group, placebo-controlled design, with six dose-escalation schemes for survodutide (BI 456906, Boehringer Ingelheim Pharma GmbH & Co. KG, Germany; electronic supplementary material [ESM] Fig. 1). The study included an open-label semaglutide group, which served as a reference control to permit comparison of response curves and support assumptions for the design of Phase III studies. Participants were assigned to one of six survodutide DGs (0.3 mg once weekly [qw; DG1], up to 0.9 mg qw [DG2], up to 1.8 mg qw [DG3], up to 2.7 mg qw [DG4], up to 1.2 mg biw [2.4 mg total; DG5] or up to 1.8 mg biw [3.6 mg total; DG6]), placebo or semaglutide (up to 1.0 mg qw). The trial was conducted in clinical research centres, including hospitals and healthcare centres. Each investigational site had a principal investigator who was responsible for the conduct of the study. See the ESM for a list of study sites and investigators.

Eligible participants were aged 18–75 years, had been diagnosed with type 2 diabetes for ≥6 months, had an HbA_1c_ value of 53–86 mmol/mol (7.0–10.0%) and a BMI of 25–50 kg/m^2^ at screening and had been treated with a stable dose of metformin of ≥1000 mg/day (immediate or extended release) for ≥3 months before screening. Exclusion criteria are listed in the ESM Methods. The full protocol is available at https://clinicaltrials.gov/ct2/show/NCT04153929.

### Randomisation and blinding

Randomisation to survodutide or placebo was in a 5:1 ratio within DGs (planned randomisation: survodutide, *n*=50 per DG; placebo, *n*=60); it was planned to randomise 50 participants to the semaglutide group. The trial was double-blind within DG1–6. Further details of randomisation and blinding are provided in the ESM Methods.

### Endpoints

The primary endpoint was the absolute change in HbA_1c_ from baseline after 16 weeks’ treatment. Secondary endpoints were related to changes in bodyweight from baseline after 16 weeks’ treatment: the relative change in bodyweight (key secondary endpoint), absolute change in bodyweight, absolute change in waist circumference and proportion of participants with a ≥5% and ≥10% decrease in bodyweight. Further efficacy endpoints are described in the ESM Methods.

Pharmacodynamic endpoints were the changes from baseline in exploratory biomarkers related to liver function and fatty liver disease (plasma levels of cytokeratin 18 and Pro-C3 and enhanced liver fibrosis [ELF] score), glucose metabolism (adiponectin and fasting insulin and C-peptide levels) and target receptor engagement (amino acid and glucagon levels). Exploratory NASH-related scores (Fibrosis-4 [Fib-4] score, aspartate aminotransferase/platelet ratio [APRI] and non-alcoholic fatty liver disease [NAFLD] fibrosis score) were assessed as safety-related endpoints.

The attainment of steady state and dose proportionality of survodutide were assessed as pharmacokinetic endpoints.

### Procedures

After completion of the 16 week treatment period, all participants had an end-of-treatment (EoT) visit (week 17). Participants then entered a 4-week follow-up period and completed the study. Details of treatment administration are provided in the ESM Methods.

HbA_1c_ was assessed at screening, weeks 1, 5, 8, 12 and 16, EoT and follow-up and analysed centrally. Bodyweight was measured at every visit (screening, weeks 1–8, 12 and 16, EoT and follow-up). Waist circumference was measured at screening, weeks 1 and 6 and EoT. Waist circumference was measured at the midpoint between the lowest rib and the iliac crest. Participants were provided with a glucose monitoring device for weekly use at home during the study. Participant-reported outcomes (Three-Factor Eating Questionnaire [TFEQ-R18 V2], Patient Global Impression of Severity [PGI-S] and a hunger visual analogue scale [VAS]) were assessed at weeks 1, 5 and 8 and EoT in a fasted state. Blood sampling for pharmacokinetics was carried out at every visit (weeks 1–8, 12 and 16, EoT and follow-up) and blood sampling for exploratory biomarkers was carried out at weeks 1, 5, 8 and 12, EoT and follow-up. Safety assessments are described in the ESM Methods.

### Statistical analyses

The trial planned to screen 615 people and randomise 410 participants at 80 study sites in multiple countries. The sample size calculation was based on an assumed maximum effect size for survodutide vs placebo of a 0.5–0.6% change (SD 1%) in HbA_1c_ for the primary endpoint, similar to that seen in a Phase II trial of semaglutide [16]. In this study, HbA_1c_ was measured in per cent. In order to report HbA_1c_ results in mmol/mol, HbA_1c_ (%) was converted to HbA_1c_ (mmol/mol) before analysis using the following equation: HbA_1c_ (mmol/mol) = 10.929 × (HbA_1c_ [%] – 2.15). Full details of the statistical analyses are provided in the ESM Methods.

### Study ethics

This trial was approved by the relevant institutional review boards, independent ethics committees and competent authorities, according to national and international regulations. The study was conducted in compliance with ethical principles laid down in the Declaration of Helsinki, in accordance with the International Council for Harmonisation Guideline for Good Clinical Practice (ICH GCP). All participants provided written informed consent, according to the ICH GCP and regulatory and legal requirements of the participating countries.

### Trial registration

This trial was registered at ClinicalTrials.gov (NCT04153929) and EudraCT (2019-002390-60).

## Results

### Study participants and compliance

Participants were recruited between 9 June 2020 and 7 June 2021; the last participant completed the trial on 5 November 2021. In total, 669 people were screened, 413 were randomised and 411 were treated (DGs 1, 2 and 4, *n*=50 each; DG3, *n*=52; DG5, *n*=51; DG6, *n*=49; semaglutide up to 1.0 mg qw, *n*=50; placebo, *n*=59; ESM Fig. 2). Of the 411 participants treated, 80 (19.5%) prematurely discontinued treatment, 53 (12.9%) owing to an AE. Important protocol deviations were reported for 62 of all randomised participants (15.0%), with two-thirds (*n*=41) due to restricted medication use. All 411 participants treated were analysed for efficacy (full analysis set: all randomised participants who received one or more dose of the study drug and had analysable data for one or more efficacy endpoint) and safety (treated set: all randomised participants who received one or more dose of the study drug). Baseline characteristics and demographics were similar between DGs (*N*=411); 83.7% of participants were White and mean ± SD age was 57.3±9.8 years, BMI 33.9±6.0 kg/m^2^ and HbA_1c_ 64.7±9.2 mmol/mol (8.1±0.8%) (Table 1). The population included in this study was representative of a large study population of people with type 2 diabetes with respect to age and HbA_1c_ levels [17]; however, most participants were White and had a higher bodyweight, due to the inclusion criteria of this trial.

**Table 1:** Participant baseline characteristics and demographics

Characteristic	DG1: Survodutide 0.3 mg qw (*n*=50)	DG2: Survodutide 0.9 mg qw (*n*=50)	DG3: Survodutide 1.8 mg qw (*n*=52)	DG4: Survodutide 2.7 mg qw (*n*=50)	DG5: Survodutide 1.2 mg biw (*n*=51)	DG6: Survodutide 1.8 mg biw (*n*=49)	Semaglutide 1.0 mg qw (*n*=50)	Placebo (*n*=59)	Total (*N*=411)
Sex									
Male	26 (52.0)	28 (56.0)	27 (51.9)	33 (66.0)	27 (52.9)	27 (55.1)	34 (68.0)	31 (52.5)	233 (56.7)
Race and ethnicity									
White	42 (84.0)	44 (88.0)	42 (80.8)	43 (86.0)	41 (80.4)	42 (85.7)	43 (86.0)	47 (79.7)	344 (83.7)
Asian	4 (8.0)	5 (10.0)	8 (15.4)	4 (8.0)	5 (9.8)	3 (6.1)	5 (10.0)	8 (13.6)	42 (10.2)
Black or African American	3 (6.0)	1 (2.0)	2 (3.8)	2 (4.0)	4 (7.8)	3 (6.1)	2 (4.0)	3 (5.1)	20 (4.9)
American Indian or Alaska Native	1 (2.0)	0	0	0	0	1 (2.0)	0	0	2 (0.5)
Native Hawaiian or Other Pacific Islander	0	0	0	0	0	0	0	1 (1.7)	1 (0.2)
Missing	0	0	0	1 (2.0)	1 (2.0)	0	0	0	2 (0.5)
Age, years	56.1±10.2	58.2±9.6	55.3±10.3	59.6±8.5	58.3±8.8	57.7±9.4	55.8±10.5	57.5±10.5	57.3±9.8
HbA_1c_, mmol/mol	64.9±8.3	62.8±8.8	65.5±9.4	65.9±10.6	65.1±10.3	63.6±7.8	64.3±9.2	65.6±9.2	64.7±9.2
HbA_1c_, %	8.09±0.76	7.89±0.80	8.14±0.86	8.18±0.97	8.11±0.94	7.97±0.71	8.03±0.82	8.15±0.85	8.07±0.84
Time from type 2 diabetes diagnosis, years	6.1±4.7	7.7±7.3	7.0±5.6	7.9±5.7	8.8±7.1	7.4±5.3	7.9±4.7	7.9±5.6	7.6±5.8
Weight, kg	97.6±19.7	100.1±19.8	95.9±22.8	96.6±22.8	95.0±22.1	98.3±24.4	96.7±20.0	93.0±21.0	96.6±21.6
BMI, kg/m^2^	33.8±6.1	34.9±5.2	33.6±5.8	34.0±6.8	33.0±5.0	34.9±7.0	33.4±6.1	33.4±5.9	33.9±6.0
Waist circumference, cm	110.6±12.8	111.5±15.6	107.2±20.0	110.7±16.4	109.0±18.2	115.1±28.7	108.1±13.5	110.4±16.5	110.3±18.2

Data are presented as *n*/*N* (%) or mean ± SD. Sex and race were self-reported

### Primary endpoint

Absolute HbA_1c_ (mixed model for repeated measures [MMRM] estimates; primary endpoint) decreased from baseline after 16 weeks’ treatment with survodutide, with a markedly weaker treatment effect observed in DG1 (0.3 mg qw) than in the other DGs; no obvious dose-dependent effects were observed between DG2–6 (Fig. 1). The results of the multiple contrast test showed that the contrasts of all predefined dose–response models were significant in terms of non-flat dose–response for the absolute change from baseline in HbA_1c_ after 16 weeks of treatment at a one-sided α=0.025. According to the final multiple comparisons procedure with modelling (MCPMod) averaging model, the predicted dose–response reached a plateau at 1.8 mg qw survodutide, with no increase in treatment effect seen at doses higher than this (ESM Fig. 3a). After 16 weeks’ treatment with survodutide, adjusted mean (95% CI) HbA_1c_ levels decreased from a baseline (mean ± SD) of 64.7±9.2 mmol/mol (8.07±0.84%; *N*=411) as follows: DG1 (*n*=41), −9.92 mmol/mol (−12.27, −7.56; −0.91% [−1.12, −0.69]); DG2 (*n*=46), −15.95 mmol/mol (−18.27, −13.63; −1.46% [−1.67, −1.25]); DG3 (*n*=36), −18.72 mmol/mol (−21.15, −16.29; −1.71% [−1.94, −1.49]); DG4 (*n*=33), −17.01 mmol/mol (−19.59, −14.43; −1.56% [−1.79, −1.32]); DG5 (*n*=44), −17.84 mmol/mol (−20.18, −15.51; −1.63% [−1.85, −1.42]); DG6 (*n*=36), −18.38 mmol/mol (−20.90, −15.87; −1.68% [−1.91, −1.45]). The decrease from baseline was significantly greater for all survodutide groups compared with placebo (−1.62 mmol/mol [−3.83, 0.59]; −0.15% [−0.35, 0.05]; *n*=49) at all tested time points (*p*<0.0001 for all DGs and time points except DG1 week 5 [*p*=0.0004]). After 16 weeks’ treatment, low-dose survodutide treatment (0.9 mg qw [DG2]) reduced HbA_1c_ to approximately the same extent as semaglutide (*n*=45) up to 1.0 mg qw ( −15.95 mmol/mol [−1.46%] vs −16.07 mmol/mol [−1.47%], respectively). Descriptive statistics of the primary endpoint revealed similar results to the MMRM analysis (ESM Fig. 3b); survodutide reduced mean ± SD HbA_1c_ by up to 19.5 mmol/mol (1.88%) in both DG3 (*n*=36) and DG6 (*n*=36) after 16 weeks, with a low dose (DG2, *n*=46) again showing similar results to the reductions seen with semaglutide (*n*=45) up to 1.0 mg qw (−14.9±10.2 mmol/mol [−1.37±0.93%] vs −16.4±9.2 mmol/mol [−1.50±0.84%], respectively).

### Secondary endpoints

**Fig. 2:**
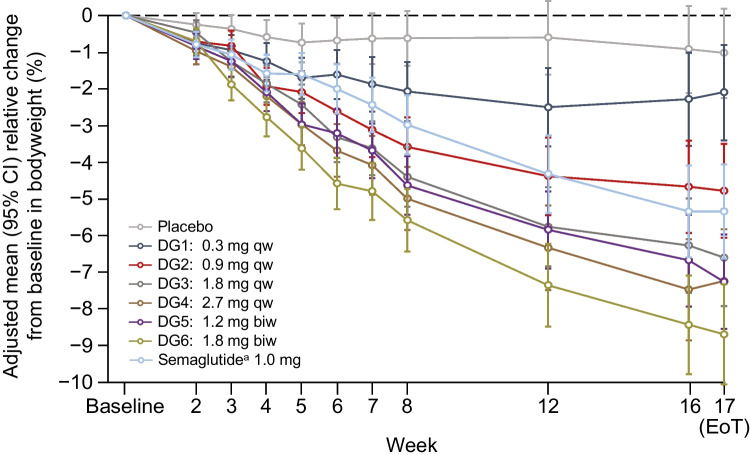
MMRM estimates for the relative change in bodyweight from baseline to EoT. ^a^Semaglutide arm was open label

The relative and absolute reduction from baseline in bodyweight was greater with increasing survodutide dose, with bodyweight loss seen in all survodutide DGs in a dose-dependent manner (Fig. 2, ESM Fig. 4). After 16 weeks of treatment, the relative decrease in bodyweight from baseline (key secondary endpoint) for DG2–6 was significantly greater than for placebo (*p*<0.001), with a maximum adjusted mean (95% CI) MMRM estimate for relative bodyweight reduction of −8.7% (−10.1, −7.3; DG6, *n*=37; Fig. 2). Survodutide doses of ≥1.8 mg qw produced greater adjusted mean (95% CI) bodyweight reductions than semaglutide up to 1.0 mg qw (DG3 [*n*=36] vs semaglutide [*n*=45]: −6.6% [−7.9, −5.3] vs –5.3% [−6.6, −4.1]). Results of the multiple contrast test showed that all predefined dose–response models were significant in terms of non-flat dose–response for the relative change from baseline in bodyweight at a one-sided α=0.025. A significant dose–response was seen in the final MCPMod averaging model and did not reach a plateau (ESM Fig. 4a). Descriptive statistics of the relative change from baseline in bodyweight were similar to the MMRM analysis (ESM Fig. 4b). The adjusted mean (95% CI) MMRM estimates for absolute changes from baseline in bodyweight after 16 weeks’ treatment were consistent with the relative changes, with favourable results seen for DG3–6 (up to −8.4 kg [−9.7, −7.1]; DG6, *n*=37) compared with semaglutide (*n*=45) up to 1.0 mg qw (−5.2 kg [−6.4, −4.0]) (ESM Fig. 4c).

Analysis of additional secondary endpoints showed that the proportion of participants with ≥5% and ≥10% reductions in bodyweight after 16 weeks’ treatment increased dose-dependently with survodutide (Table 2). In DG6, 57.1% of participants (*n*=28) had ≥5% and 34.7% (*n*=17) had ≥10% bodyweight reductions; this compares with 6.8% (*n*=4) and 0%, respectively, for placebo and 38.0% (*n*=19) and 16.0% (*n*=8), respectively, for semaglutide up to 1.0 mg qw. The probability of achieving ≥5% or ≥10% bodyweight loss was significantly greater in DG2–6 for ≥5% loss (OR [95% CI] DG2, 7.92 [2.43, 25.74]; DG3, 17.68 [5.21, 60.03]; DG4, 25.87 [7.31, 91.55]; DG5, 21.75 [6.57, 72.04]; DG6, 35.00 [9.84, 124.47]) and DG3–6 for ≥10% loss (DG3, 25.17 [1.35, 470.86]; DG4, 33.00 [1.78, 613.20]; DG5, 42.43 [2.37, 761.05]; DG6, 84.51 [4.71, >999]) compared with placebo (Table 2).

**Table 2:** Proportion of participants achieving ≥5% and ≥10% bodyweight reductions

Bodyweight reduction	DG1: Survodutide 0.3 mg qw (*n*=50)	DG2: Survodutide 0.9 mg qw (*n*=50)	DG3: Survodutide 1.8 mg qw (*n*=52)	DG4: Survodutide 2.7 mg qw (*n*=50)	DG5: Survodutide 1.2 mg biw (*n*=51)	DG6: Survodutide 1.8 mg biw (*n*=49)	Semaglutide 1.0 mg qw (*n*=50)	Placebo (*n*=59)
≥5%, *n* (%)	4 (8.0)	19 (38.0)	22 (42.3)	23 (46.0)	29 (56.9)	28 (57.1)	19 (38.0)	4 (6.8)
Vs placebo								–
OR	1.22	7.92	17.68	25.87	21.75	35.00	8.22	–
(95% CI)	(0.28, 5.20)	(2.43, 25.74)	(5.21, 60.03)	(7.31, 91.55)	(6.57, 72.04)	(9.84, 124.47)	(2.52, 26.79)	–
≥10%, *n* (%)	1 (2.0)	3 (6.0)	7 (13.5)	8 (16.0)	13 (25.5)	17 (34.7)	8 (16.0)	0
Vs placebo								–
OR	3.67	7.97	25.17	33.00	42.43	84.51	22.44	–
(95% CI)	(0.14, 95.68)	(0.39, 163.48)	(1.35, 470.86)	(1.78, 613.20)	(2.37, 761.05)	(4.71, >999)	(1.22, 413.13)	–

Waist circumference decreased from baseline with both survodutide and semaglutide treatment, but data were highly variable and associated with wide 95% CIs (ESM Fig. 5). The maximum adjusted mean ± SEM MMRM estimate for decrease in waist circumference from baseline was observed after 16 weeks’ treatment in DG6 (−10.5±1.7 cm; *n*=36). Adjusted mean ± SEM MMRM estimates for the placebo-corrected absolute change from baseline after 16 weeks’ treatment were significant for DG4 (−4.6±2.25 cm; *n*=35; *p*=0.041) and DG6 (−8.4±2.24 cm; *n*=36; *p*=0.0002).

### Further efficacy endpoints

**Fig. 3:**
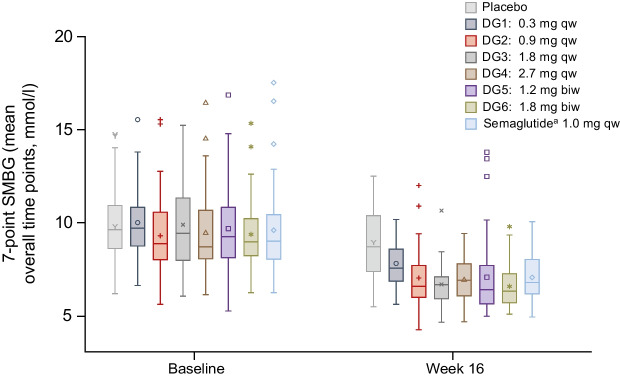
Change from baseline in 7-point SMBG. Blood glucose measurements were collected before each meal (assuming three meals a day), approximately 2 h after each meal and at bedtime on a single day during screening (baseline) and a single day between the last dose of study drug and EoT (week 16). Mean overall time points represent the mean per participant per visit of the seven blood glucose measurements at baseline and week 16. Data are presented per DG. The centre line denotes the median value, with the symbols within the boxes denoting the mean. The box boundaries mark the upper and lower quartile of the dataset. The whiskers indicate the variability of the data; whiskers are drawn to the nearest value within 1.5× the IQR of the upper and lower quartiles. Any observations outside of these values are plotted with symbols. ^a^Semaglutide arm was open label

Treatment with survodutide or semaglutide reduced the mean 7-point self-monitoring of blood glucose (SMBG) level to a greater extent than placebo at week 16; the maximum mean ± SD decrease from baseline was observed in DG3 (−3.03±2.47 mmol/l; *n*=36) (Fig. 3), and decreases were more pronounced at post-mealtime time points than at pre-mealtime time points.

Minor treatment effects were observed for all domains of the TFEQ-R18 V2, hunger VAS and PGI-S; a full description of these results is given in the ESM for the 16 week time frame (ESM Tables 1 and 2).

### Safety

A total of 77.8% of participants (*n*=235) treated with survodutide reported at least one treatment-emergent adverse event (TEAE); 52.5% of those receiving placebo (*n*=31) and 52.0% receiving semaglutide (*n*=26) also reported TEAEs (Table 3). Of these, severe AEs were reported by 16 participants treated with survodutide (5.3%), four receiving placebo (6.8%) and none receiving semaglutide; these were mostly GI disorders (*n*=8/16 [50%] for survodutide; *n*=1/4 [25%] for placebo). Serious AEs were reported by 3.6% of participants receiving survodutide (*n*=11: DG1, *n*=1; DG2, *n*=4; DG3, *n*=3; DG4, *n*=2; DG5, *n*=1) and 5.1% receiving placebo (*n*=3) (Table 3, ESM Table 3). AEs led to treatment discontinuation in 15.9% of participants receiving survodutide (*n*=48), 5.1% receiving placebo (*n*=3) and 4.0% receiving semaglutide (*n*=2). Most of these discontinuations occurred within the first 6 weeks of the study (*n*=38/53, 71.7%), coinciding with the dose-escalation period, and were mainly due to GI disorders (survodutide: *n*=36/48, 75.0%; placebo *n*=1/3, 33.3%; semaglutide: 0) such as nausea and vomiting. More discontinuations due to AEs were observed in those with baseline bodyweight <100 kg than those with bodyweight ≥100 kg. GI disorders were the most frequently reported AEs across all DGs, occurring in 55.3% of participants receiving survodutide, 22.0% receiving placebo and 28.0% receiving semaglutide.

**Table 3:** Summary of AEs

AE	DG1: Survodutide 0.3 mg qw (*n*=50)	DG2: Survodutide 0.9 mg qw (*n*=50)	DG3: Survodutide 1.8 mg qw (*n*=52)	DG4: Survodutide 2.7 mg qw (*n*=50)	DG5: Survodutide 1.2 mg biw (*n*=51)	DG6: Survodutide 1.8 mg biw (*n*=49)	Semaglutide 1.0 mg qw (*n*=50)	Placebo (*n*=59)	Total survodutide (*n*=302)
Any TEAE	33 (66.0)	38 (76.0)	42 (80.8)	41 (82.0)	39 (76.5)	42 (85.7)	26 (52.0)	31 (52.5)	235 (77.8)
Investigator-defined, drug-related AEs^a^	25 (50.0)	26 (52.0)	33 (63.5)	29 (58.0)	28 (54.9)	36 (73.5)	19 (38.0)	13 (22.0)	177 (58.6)
Nausea	10 (20.0)	13 (26.0)	24 (46.2)	20 (40.0)	14 (27.5)	22 (44.9)	6 (12.0)	5 (8.5)	103 (34.1)
Vomiting	6 (12.0)	7 (14.0)	12 (23.1)	13 (26.0)	6 (11.8)	10 (20.4)	2 (4.0)	1 (1.7)	54 (17.9)
Diarrhoea	11 (22.0)	5 (10.0)	8 (15.4)	7 (14.0)	8 (15.7)	9 (18.4)	4 (8.0)	5 (8.5)	48 (15.9)
Dyspepsia	4 (8.0)	3 (6.0)	3 (5.8)	4 (8.0)	3 (5.9)	6 (12.2)	1 (2.0)	0	23 (7.6)
Decreased appetite	6 (12.0)	7 (14.0)	5 (9.6)	9 (18.0)	8 (15.7)	15 (30.6)	3 (6.0)	2 (3.4)	50 (16.6)
Headache	2 (4.0)	5 (10.0)	3 (5.8)	0	3 (5.9)	2 (4.1)	0	1 (1.7)	15 (5.0)
Severe AEs^b^	3 (6.0)	1 (2.0)	4 (7.7)	3 (6.0)	2 (3.9)	3 (6.1)	0	4 (6.8)	16 (5.3)
Nausea	1 (2.0)	0	1 (1.9)	0	0	1 (2.0)	0	0	3 (1.0)
Vomiting	2 (4.0)	0	0	0	2 (3.9)	0	0	0	4 (1.3)
Dizziness	0	0	0	0	0	2 (4.1)	0	0	2 (0.7)
Serious AEs	1 (2.0)	4 (8.0)	3 (5.8)	2 (4.0)	1 (2.0)	0	0	3 (5.1)	11 (3.6)
Drug-related serious AEs	1 (2.0)	1 (2.0)	1 (1.9)	1 (2.0)	0	0	0	0	4 (1.3)
AEs leading to treatment discontinuation	5 (10.0)	5 (10.0)	11 (21.2)	15 (30.0)	4 (7.8)	8 (16.3)	2 (4.0)	3 (5.1)	48 (15.9)

Data are presented as *n* (%)

^a^Drug-related AEs reported by preferred term in ≥10% of participants

^b^Severe AEs reported by preferred term in two or more participants in all survodutide DGs

Of participants treated with survodutide, 58.6% (*n*=177) reported drug-related AEs, compared with 22.0% receiving placebo (*n*=13) and 38.0% of those receiving semaglutide (*n*=19) (Table 3). The majority of AEs were GI disorders (nausea, vomiting, diarrhoea and dyspepsia), which were reported for 50.0% (*n*=151) receiving survodutide. Drug-related AEs were classed as serious for 1.3% of participants receiving survodutide (*n*=4); DG1 (abdominal pain and vomiting), DG2 (mouth ulceration, autoimmune disorder and pharyngeal ulceration), DG3 (dehydration) and DG4 (diarrhoea) (*n*=1 each; Table 3, ESM Table 3).

Mean heart rate was slightly increased from baseline in all treatment groups; the mean increase after 16 weeks was 2.3–7.3 beats per min (bpm) across the survodutide DGs, 5.9 bpm for semaglutide and 1.67 bpm for placebo. The mean increases in heart rate from baseline were below 10 bpm at all time points except for two in DG4 (10.3 bpm at week 7; 10.1 bpm at week 16). There were no new onsets reported in the QTcF (QT interval corrected for heart rate using the method of Fridericia) interval categories >480–500 msec or >500 msec; one participant each from DG3 (2.0%) and the placebo group (1.7%) reported an increase in QTcF interval of >60 msec. The changes from baseline in QTcF interval were considered minor and no increased risk of cardiovascular events was identified.

### Pharmacodynamic endpoints

Treatment with survodutide did not lead to any clear dose-dependent reductions in the NASH-related Fib-4 score, APRI and NAFLD fibrosis score relative to placebo (Table 4). Mean ± SD Pro-C3 levels were substantially lowered from baseline over time in all survodutide DGs (up to −7.3±9.4 µg/l in DG5; *n*=43) and the semaglutide group (−5.2±7.3 µg/l; *n*=44) compared with placebo (−0.1±10.2 µg/l; *n*=48) (Table 4). Changes from baseline in ELF score were detected in all treatment groups, with decreases observed in DG2–6, up to a mean ± SD change of −0.2±0.5 in DG6 (*n*=36) compared with an increase of 0.2±0.4 in the placebo group (*n*=49) (Table 4). The changes from baseline in other exploratory biomarkers are presented in Table 4.

**Table 4:** Absolute change from baseline in exploratory variables at EoT

Exploratory variable	DG1: Survodutide 0.3 mg qw (*n*=50)	DG2: Survodutide 0.9 mg qw (*n*=50)	DG3: Survodutide 1.8 mg qw (*n*=52)	DG4: Survodutide 2.7 mg qw (*n*=50)	DG5: Survodutide 1.2 mg biw (*n*=51)	DG6: Survodutide 1.8 mg biw (*n*=49)	Semaglutide 1.0 mg qw (*n*=50)	Placebo (*n*=59)
Fasting C-peptide, nmol/l	0.134 ± 0.367 (*n*=41)	0.065 ± 0.350 (*n*=47)	0.035 ± 0.450 (*n*=37)	0.214 ± 0.709 (*n*=33)	0.109 ± 0.429 (*n*=44)	0.073 ± 0.468 (*n*=37)	0.111 ± 0.322 (*n*=44)	0.033 ± 0.250 (*n*=49)
Fasting C-peptide, μg/l	0.405 ± 1.113 (*n*=41)	0.196 ± 1.059 (*n*=47)	0.105 ± 1.364 (*n*=37)	0.648 ± 2.149 (*n*=33)	0.331 ± 1.300 (*n*=44)	0.222 ± 1.417 (*n*=37)	0.337 ± 0.977 (*n*=44)	0.101 ± 0.758 (*n*=49)
Fasting insulin, pmol/l	29.000 ± 98.562 (*n*=40)	1.328 ± 62.680 (*n*=45)	15.637 ± 102.041 (*n*=37)	33.645 ± 149.897 (*n*=31)	13.263 ± 78.773 (*n*=43)	16.670 ± 96.480 (*n*=37)	1.553 ± 72.655 (*n*=44)	11.176 ± 44.242 (*n*=48)
Plasma glucagon, ng/l	–15.85 ± 27.47 (*n*=28)	–20.52 ± 41.12 (*n*=32)	–34.06 ± 42.84 (*n*=26)	–29.67 ± 46.99 (*n*=24)	–38.15 ± 40.38 (*n*=32)	–52.51 ± 55.53 (*n*=22)	–18.05 ± 33.44 (*n*=30)	–13.20 ± 31.07 (*n*=34)
Pro-C3, μg/l	–1.91 ± 7.12 (*n*=42)	–3.46 ± 5.35 (*n*=45)	–6.84 ± 8.89 (*n*=36)	–6.82 ± 7.43 (*n*=33)	–7.27 ± 9.37 (*n*=43)	–5.74 ± 7.31 (*n*=36)	–5.21 ± 7.34 (*n*=44)	–0.06 ± 10.18 (*n*=48)
ELF score	0.056 ± 0.503 (*n*=40)	–0.030 ± 0.482 (*n*=47)	–0.095 ± 0.550 (*n*=37)	–0.136 ± 0.610 (*n*=33)	–0.013 ± 0.583 (*n*=44)	–0.167 ± 0.455 (*n*=36)	0.017 ± 0.622 (*n*=44)	0.193 ± 0.443 (*n*=49)
HMW adiponectin, μg/l	–244.4 ± 1619.0 (*n*=41)	235.9 ± 1549.2 (*n*=45)	211.3 ± 899.6 (*n*=37)	343.8 ± 1128.3 (*n*=32)	281.9 ± 1486.1 (*n*=44)	649.1 ± 2089.1 (*n*=36)	80.0 ± 1171.5 (*n*=44)	392.9 ± 1929.7 (*n*=49)
CK-18 (M30), U/l	–46.93 ± 123.16 (*n*=41)	–98.36 ± 201.33 (*n*=46)	–101.57 ± 189.65 (*n*=36)	–74.47 ± 116.55 (*n*=32)	–141.93 ± 233.62 (*n*=43)	–98.36 ± 161.29 (*n*=35)	–135.20 ± 227.74 (*n*=44)	–44.68 ± 160.64 (*n*=49)
CK-18 (M65), U/l	–25.93 ± 143.14 (*n*=41)	–154.17 ± 307.29 (*n*=46)	–187.07 ± 343.71 (*n*=37)	–120.06 ± 178.13 (*n*=32)	–198.79 ± 316.00 (*n*=44)	–208.24 ± 321.33 (*n*=36)	–207.16 ± 322.21 (*n*=44)	–101.55 ± 263.48 (*n*=49)
Fib-4 score	0.029 ± 0.264 (*n*=40)	–0.080 ± 0.328 (*n*=46)	–0.145 ± 0.299 (*n*=36)	–0.064 ± 0.306 (*n*=33)	–0.157 ± 0.194 (*n*=44)	–0.185 ± 0.429 (*n*=32)	–0.026 ± 0.229 (*n*=43)	–0.027 ± 0.239 (*n*=44)
APRI	–0.018 ± 0.106 (*n*=40)	–0.046 ± 0.108 (*n*=46)	–0.094 ± 0.135 (*n*=36)	–0.055 ± 0.088 (*n*=33)	–0.078 ± 0.094 (*n*=44)	–0.096 ± 0.214 (*n*=32)	–0.041 ± 0.095 (*n*=43)	–0.014 ± 0.060 (*n*=44)
NAFLD fibrosis score	0.126 ± 0.595 (*n*=40)	–0.052 ± 0.667 (*n*=46)	–0.090 ± 0.670 (*n*=36)	0.097 ± 0.604 (*n*=33)	–0.162 ± 0.659 (*n*=44)	–0.032 ± 0.586 (*n*=32)	0.004 ± 0.541 (*n*=43)	0.049 ± 0.649 (*n*=44)

Data are presented as mean ± SD

CK-18, cytokeratin 18; HMW, high molecular weight; M30, caspase-cleaved cytokeratin 18; M65, full-length cytokeratin 18 (including caspase cleaved and intact)

Plasma exposure to survodutide increased with escalating weekly or biweekly doses, with trough concentrations increasing in an approximately dose-proportional manner (ESM Fig. 6). Visually, steady state for survodutide appeared to be achieved at week 8 for DG1–5 and at week 12 for DG6 (ESM Fig. 6).

Glucagon levels decreased dose-dependently from baseline over 16 weeks’ treatment with survodutide, suggesting target engagement of GCGRs and GLP-1Rs (ESM Fig. 7a). The mean ± SEM changes after 16 weeks were most pronounced in DG5 and DG6 (−11.0±2.0 pmol/l [*n*=32] and −15.1±3.4 pmol/l [*n*=22], respectively), and no relevant changes from baseline were observed in the semaglutide or placebo groups. Although small treatment effects on plasma amino acid levels were observed in DG6 compared with semaglutide up to 1.0 mg qw and placebo following 16 weeks’ treatment, these were only notable for alanine; the mean ± SEM decrease in alanine from baseline to EoT in DG6 (−58.4±24.2 µmol/l; *n*=36) was indicative of target engagement at GCGRs (ESM Fig. 7b).

## Discussion

GLP-1R agonists such as semaglutide have been approved for the treatment of type 2 diabetes and obesity [18]. Recent data on the efficacy of tirzepatide demonstrate that incretin dual agonists have the potential to more effectively reduce HbA_1c_ and bodyweight than GLP-1R agonists alone [5]. Therefore, development of incretin dual agonists such as the novel GCGR/GLP-1R dual agonist survodutide is an important step towards the more effective treatment of people with type 2 diabetes and obesity. In this study, survodutide dose-dependently reduced HbA_1c_ after 16 weeks’ treatment (by up to −18.72 mmol/mol [−1.71%]). The efficacy of survodutide was compared with open-label semaglutide (up to 1.0 mg qw); survodutide was shown to be equally efficacious at lowering HbA_1c_ at low doses (−1.46% for DG2 vs −1.47% for semaglutide). Furthermore, survodutide at doses ≥1.8 mg qw induced greater bodyweight reductions than semaglutide after 16 weeks’ treatment (up to −8.7% [8.4 kg] DG6 vs −5.3% [5.2 kg] semaglutide).

In addition to semaglutide, other GLP-1R agonists now approved for the treatment of type 2 diabetes have been investigated in combination with background metformin therapy. In the LEAD-2 study, liraglutide treatment for 26 weeks produced HbA_1c_ reductions of up to –10.9 mmol/mol (–1.0%) and bodyweight reductions of up to –2.8 kg from baseline [19]. These results are relatively similar to those seen with dulaglutide in the AWARD-5 study, which produced reductions of –12.0 mmol/mol (−1.1%) and –3.1 kg for HbA_1c_ and bodyweight, respectively, after 52 weeks of treatment [20]. The maximum reductions in HbA_1c_ and bodyweight observed in the current study (−18.72 mmol/mol [−1.71%] and −8.4 kg, respectively) exceeded those of the above studies, after a shorter treatment duration of only 16 weeks.

Furthermore, a Phase II dose-finding study of the weekly GIPR/GLP-1R dual agonist tirzepatide found reductions in HbA_1c_ and bodyweight that were in line with the results of the present study; tirzepatide treatment up to 15 mg qw reduced HbA_1c_ by up to −21.9 mmol/mol (−2.0%) and bodyweight by up to −5.7 kg after 12 weeks [21]. This highlights the suggested additional efficacy of incretin dual agonists over GLP-1R mono-agonists.

Another GCGR/GLP-1R dual agonist, cotadutide, has been developed for the treatment of type 2 diabetes and obesity. In a Phase IIa trial in participants receiving metformin background therapy, cotadutide treatment for 49 days led to a significant reduction in blood glucose (*p*<0.001) and bodyweight reductions of –3.41% from baseline vs placebo [22]. A Phase IIb study comparing cotadutide with the GLP-1R mono-agonist liraglutide showed that, after 54 weeks, cotadutide produced similar HbA_1c_ reductions (cotadutide vs liraglutide: −13.0 mmol/mol vs −12.8 mmol/mol [−1.19% vs −1.17%]) but greater bodyweight reductions than liraglutide (−5.02% vs −3.33%) [6]. Although these studies also showed the potential of dual GCGR/GLP-1R agonism, the current study of survodutide produced greater HbA_1c_ and bodyweight reductions after a shorter treatment duration than the Phase II studies of cotadutide, again highlighting the potential greater therapeutic efficacy of survodutide.

The overall tolerability profile of survodutide was as expected for an incretin dual agonist. Most of the reported TEAEs with survodutide treatment were GI disorders such as nausea; drug-related AEs were reported by 58.6% of those receiving survodutide, with half of these participants reporting drug-related GI disorders. The occurrence of GI disorders is common in people treated with GLP-1R agonists [23, 24] and can be linked to the central effects of GLP-1R agonism and delayed gastric emptying [25, 26]. Although participants receiving semaglutide up to 1.0 mg qw reported fewer AEs than those receiving survodutide, these were also primarily GI-related AEs (*n*=14/26, 53.8%). The lower overall occurrence may be linked to the slower dose-escalation scheme for those in the semaglutide group, per approved prescribing information. Most of the AEs observed in participants receiving survodutide occurred during the rapid dose-escalation period of the study, and therefore the frequency of AEs (particularly GI AEs) may be mitigated by the implementation of a slower escalation scheme in future studies. In addition, the proportion of participants discontinuing treatment due to AEs was higher in those receiving survodutide than in those receiving placebo or semaglutide (15.9% vs 5.1% vs 4.0%, respectively). The discontinuations in the survodutide groups were again mostly due to GI AEs (nausea and vomiting) that occurred during the rapid dose-escalation phase. This is in line with Phase II study results for semaglutide treatment in participants with type 2 diabetes, with 11% of total participants withdrawing from the trial due to AEs (vs 12.9% in the present study), primarily due to GI AEs in the first month of treatment [16]. The results of the Phase II semaglutide study also highlighted that the proportion of participants reporting GI AEs was notably reduced with dose escalation compared with no escalation and that GI AEs were mostly transient; it was suggested from this that GI AEs may be ameliorated with slower dose escalation [16]. This supports the suggestion that slower escalations of survodutide over a longer treatment period should be explored in future trials to mitigate the occurrence of GI AEs.

The observed changes in plasma alanine and glucagon concentrations with survodutide but not placebo or semaglutide indicate target engagement at both the GCGR and the GLP-1R, highlighting that survodutide is a GCGR/GLP-1R dual agonist and its effects are exerted at both receptors. Amino acids are sensitive biomarkers for assessing GCGR activity of GCGR/GLP-1R dual agonists, as plasma amino acid levels are reduced independently of insulin and glucose levels and GLP-1R activity [27, 28]. Both GLP-1R and GCGR agonism can reduce levels of plasma glucagon, as seen in preclinical studies of survodutide [14], and therefore glucagon acts as a marker for both GLP-1R and GCGR activation. GCGR agonism can increase energy expenditure and stimulate hepatic glucose production, lipolysis, amino acid breakdown and suppression of hepatic fat accumulation. The addition of GCGR agonism to GLP-1R agonism may therefore prove to be more efficacious in the treatment of type 2 diabetes and obesity, producing additive effects on energy intake and expenditure.

GCGR/GLP-1R dual agonism is also a focus in the treatment of NASH as it can potentially ameliorate hepatic fat accumulation, steatosis and fibrosis, alongside reductions in bodyweight [29, 30]. In our short-term study, small reductions after survodutide treatment were observed in NASH-related scores (Fib-4 score, APRI, NAFLD fibrosis score) and in the ELF score, and potentially clinically relevant reductions were observed in the fibrogenic biomarker Pro-C3. During fibrosis, type III collagen is synthesised and deposited; therefore, Pro-C3 as the pro-peptide of type III collagen may be a useful biomarker for fibrogenesis and the monitoring of disease progression in NASH [31]. These results, particularly the reduction in Pro-C3, suggest that collagen synthesis during fibrogenesis may be suppressed with survodutide treatment, supporting the development of this therapy for the treatment of NASH.

The 16 week duration of this trial meant that rapid dose escalation of survodutide over 6 weeks was required to allow for 10 weeks of treatment and drug exposure with the maintenance dose in each DG. This escalation most likely led to a higher incidence of AEs and treatment discontinuations due to AEs during the dose-escalation phase. In future studies, more gradual dose escalations over a longer escalation phase and treatment duration may help to mitigate the occurrence of dose-related GI AEs. In addition, future studies are expected to include participants with a higher baseline bodyweight than in the current study, as discontinuations due to AEs were more frequent in participants with a baseline bodyweight of <100 kg. As a proof-of-concept, first-in-patient trial in people with type 2 diabetes, the inclusion/exclusion criteria were more restrictive and therefore the results may not be generalisable to the entire insulin-naive type 2 diabetes population. In addition, the majority of the participants were White, which may also impact the generalisability of the results. Following the promising results of this study, future studies of survodutide will allow for the use of additional concomitant medications (in addition to metformin) and include participants with additional complications.

### Conclusions

Despite the rapid dose escalation, no unexpected safety or tolerability concerns were raised and, importantly, treatment with survodutide produced greater HbA_1c_ and bodyweight reductions than semaglutide 1.0 mg qw after 16 weeks of treatment. The results of this trial highlight the potential of the novel GCGR/GLP-1R dual agonist survodutide for the treatment of NASH, type 2 diabetes and obesity.

### Supplementary Information

Below is the link to the electronic supplementary material.

Supplementary file1 (PDF 807 KB)

## Data Availability

To ensure independent interpretation of clinical study results and enable authors to fulfil their role and obligations under the ICMJE criteria, Boehringer Ingelheim grants all external authors access to relevant clinical study data. In adherence with the Boehringer Ingelheim Policy on Transparency and Publication of Clinical Study Data, scientific and medical researchers can request access to clinical study data after publication of the primary manuscript and secondary analyses in peer-reviewed journals and regulatory and reimbursement activities are completed, normally within 1 year after the marketing application has been granted by major regulatory authorities. Researchers should use the https://vivli.org/ link to request access to study data and visit https://www.mystudywindow.com/msw/datasharing for further information.

## Abbreviations

AE: Adverse event
APRI: Aspartate aminotransferase to platelet ratio
biw: Twice weekly
bpm: Beats per min
DG: Dose group
ELF: Enhanced liver fibrosis
EoT: End of treatment
Fib-4: Fibrosis-4
GCGR: Glucagon receptor
GI: Gastrointestinal
GIPR: Glucose-dependent insulinotropic polypeptide receptor
GLP-1R: Glucagon-like peptide-1 receptor
MCPMod: Multiple comparisons procedure with modelling
MMRM: Mixed model for repeated measures
NAFLD: Non-alcoholic fatty liver disease
NASH: Non-alcoholic steatohepatitis
PGI-S: Patient Global Impression of Severity
qw: Once weekly
SMBG: Self-monitoring of blood glucose
TEAE: Treatment-emergent adverse event
TFEQ-R18 V2: Three-Factor Eating Questionnaire
VAS: Visual analogue scale

## Authors’ relationships and activities

RM is an employee of Boehringer Ingelheim Pharmaceuticals, Inc. AMH is an employee of Boehringer Ingelheim International GmbH. JH is an employee of Staburo GmbH, on behalf on Boehringer Ingelheim Pharma GmbH & Co. JR has served on advisory panels for Applied Therapeutics, Boehringer Ingelheim, Eli Lilly, Hanmi, Intarcia, Novo Nordisk, Oramed, Sanofi, Structure Therapeutics, Terns Pharma and Zealand; has received honoraria for lectures sponsored by Boehringer Ingelheim, Eli Lilly, Novo Nordisk and Sanofi; and has received research support from Applied Therapeutics, AstraZeneca, Boehringer Ingelheim, Eli Lilly, Genentech, GlaxoSmithKline, Hanmi, Intarcia, Lexicon, Merck, Novartis, Novo Nordisk, Oramed, Pfizer and Sanofi. MB received honoraria as a consultant and speaker from Amgen, AstraZeneca, Bayer, Boehringer Ingelheim, Lilly, Novartis, Novo Nordisk, Pfizer and Sanofi; and has participated on a data safety monitoring board/advisory board for Boehringer Ingelheim.
