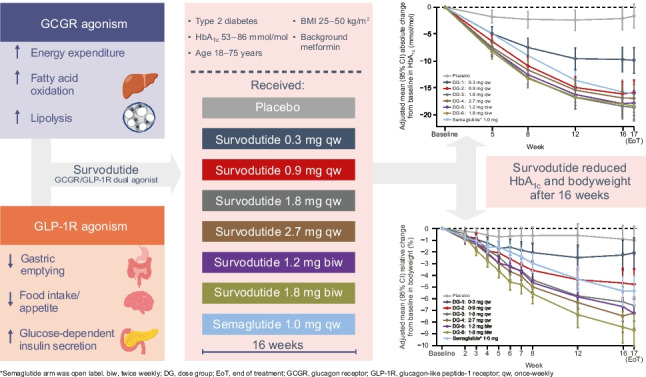# Correction to: Dose–response effects on HbA_1c_ and bodyweight reduction of survodutide, a dual glucagon/GLP-1 receptor agonist, compared with placebo and open-label semaglutide in people with type 2 diabetes: a randomised clinical trial

**DOI:** 10.1007/s00125-024-06095-7

**Published:** 2024-02-13

**Authors:** Matthias Blüher, Julio Rosenstock, Josef Hoefler, Raymond Manuel, Anita M. Hennige

**Affiliations:** 1 Helmholtz Institute for Metabolic, Obesity and Vascular Research (HI‑MAG) of the Helmholtz Zentrum München, University of Leipzig and University Hospital Leipzig, Leipzig, Germany; 2 Velocity Clinical Research, Medical City, Dallas, TX USA; 3 Staburo GmbH, Munich, Germany, on behalf of Boehringer Ingelheim Pharma GmbH & Co. KG, Biberach an der Riß, Germany; 4 Boehringer Ingelheim Pharmaceuticals, Inc., Ridgefield, CT USA; 5 Boehringer Ingelheim International GmbH, Biberach an der Riß, Germany

**Correction: Diabetologia**

10.1007/s00125-023-06053-9

The graphical abstract (available at 10.1007/s00125-023-06053-9) included a typographical error (‘insulin-dependent insulin secretion’, rather than ‘glucose-dependent insulin secretion’). The online version has been corrected.