# Supplementary material for: Dose–response effects on HbA1c and bodyweight reduction of survodutide, a dual glucagon/GLP-1 receptor agonist, compared with placebo and open-label semaglutide in people with type 2 diabetes: a randomised clinical trial
Source: Diabetologia. 2023 Dec 14;67(3):470–82. doi: 10.1007/s00125-023-06053-9 (PMC10844353; doi:10.1007/s00125-023-06053-9)
Supplement: Supplementary file 1 — Supplementary file1 (PDF 807 KB) [file 125_2023_6053_MOESM1_ESM.pdf]

Electronic supplementary material (ESM)

ESM – Methods

Exclusion criteria

Participants were excluded if they had type 1 diabetes; had been exposed to semaglutide or another glucagon-like peptide-1 receptor agonist (including combination products), or to oral antihyperglycaemic medication other than metformin in the 3 months before screening; had received treatment with insulin during the 12 months before screening; had >1 episode of ketoacidosis or hyperosmolar state requiring hospitalisation, ≥1 episode of severe hypoglycaemia (requiring the assistance of another person) or any history of hypoglycaemia unawareness or poor recognition within the 6 months prior to screening; had resting heart rate ≥100 beats per minute (bpm), blood pressure ≥160/95 mmHg, renal artery stenosis or evidence of labile blood pressure (including symptomatic hypotension or a history of orthostatic hypotension, fainting spells or blackouts) at screening; had a marked prolongation of QT/QTc (Fridericia) interval >450 ms at screening or randomisation, or any other abnormal or clinically significant ECG finding at screening (e.g. Type II Mobitz II or third-degree atrioventricular [AV] block); had a heart rhythm disturbance (e.g. bradycardia with baseline heart rate <50 bpm in the absence of medications to lower the heart rate, supraventricular tachycardia or tachyarrhythmia) considered by the investigator as indicative of relevant cardiac disease, or with abnormalities that may interfere with the interpretation of ECG intervals at screening, or had a family history of long QT-syndrome, use of prescription or over-the-counter medications known to significantly prolong the QT or QTc interval at screening; had myocardial infarction, unstable angina, clinically relevant coronary artery disease, coronary artery bypass graft, urgent percutaneous coronary intervention, transient ischaemic attack, cerebrovascular accident (stroke) or decompensated heart failure within the last 6 months before screening; had a history of or were currently diagnosed with congestive

heart failure (New York Heart Association class III–IV), or had a history of symptomatic ventricular tachycardia, history of type 2 second degree AV block or third-degree AV block; had a bodyweight change of ± 5% or more in the past 3 months or on anti-obesity therapy at any time during the 6 months before screening; ongoing oral pharmacotherapy treatments or within 3 months before screening (e.g. antidepressants, central nervous system stimulants, antipsychotics, anticonvulsants [except gabapentin], diuretics); had major surgery within 12 weeks before randomisation or planned within 6 months after screening; had prior surgery of the gastrointestinal tract that could interfere with bodyweight (except appendectomy, simple hernia repair and simple cholecystectomy); had known significant autonomic neuropathy; had acute proliferative diabetic retinopathy or expected medical intervention/surgery during the trial; had a history of chronic or acute pancreatitis or elevation of serum lipase/amylase >2 × upper limit of normal (ULN) or serum triglyceride levels >500 mg/dl at screening (doses for lipid-lowering medication must be stable for 3 months before screening); had an eGFR of <45 ml/min/1.73 m^2, or had eGFR or a serum creatinine level that would contraindicate the use of metformin; had calcitonin ≥20 pg/ml at screening; had symptoms of liver disease, chronic or acute hepatitis or serum levels of alanine aminotransferase or aspartate aminotransferase >2.5 × ULN at screening; had a personal or familial history of medullary thyroid carcinoma or history of multiple endocrine neoplasia syndrome type 2, acromegaly, morbus Cushing, or pheochromocytoma, manifest hypo- or hyperthyroidism at screening; received continuous oral pharmacotherapy to treat any clinical condition during the trial (except metformin, antihypertensives, hormone replacement therapy, lipid-lowering, proton pump inhibitors, H2 blockers for gastroesophageal reflux disease, analgesics, sleep medications, antihistamines, selective alpha receptor blocker for benign prostatic hyperplasia); used drugs with narrow therapeutic index; had a disease of the central nervous system, or other relevant neurological or psychiatric disorder; had any suicidal behaviour in

the prior 2 years or any suicidal ideation of type 4 or 5 in the Columbia-Suicide Severity Rating Scale (C-SSRS) in the 3 months before screening; had a chronic or relevant acute infection; had a history of atopy or clinically significant multiple or severe drug allergies, or intolerance to topical corticosteroids, or severe post-treatment hypersensitivity reactions; had a history or organ transplant (other than corneal transplants); abused alcohol, drugs or liquorice within the 3 months before screening; had donated ≥450 ml of blood within 3 months before screening; had a blood transfusion or severe blood loss within 3 months before screening, or had known haemoglobinopathy, haemolytic anaemia, sickle cell anaemia, or a haemoglobin value <11 or <10 g/dl (for males and females, respectively); had confirmed active infection with SARS-CoV-2 within the 3 months before screening; or were women who were pregnant, nursing or planned to become pregnant during the trial.

Randomisation and blinding

Randomisation lists were generated using interactive response technology (IRT), which involved a pseudorandom number generator so that the resulting treatment was reproducible and nonpredictable. Patients, investigators and everyone involved in the trial conduct or analysis remained blinded to the randomised treatment assignments until after database lock. Emergency unblinding was available to the investigator via IRT and was to be used only when identity of the study drug had to be known by the investigator to provide appropriate medical treatment or ensure safety of the study participants.

Endpoints

Further efficacy endpoints included the change from baseline in: the control of eating behaviour and susceptibility to hunger as measured by the change in Three-Factor Eating Questionnaire (TFEQ-R18V2), the perception of hunger by the hunger visual analogue scale

(VAS), the Patient's Global Impression of Severity (PGI-S) for difficulty to control eating behaviours and the 7-point profile of self-monitoring of blood glucose (SMBG).

Procedures

At each study visit, participants were advised to adhere to the specific recommended diet and exercise plan provided to them during diet and exercise counselling throughout the study.

Survodutide was made available in vials (2 mg/ml, 1 ml fill volume per vial) for dose escalation in Weeks 1–6 and in pre-filled syringes (various concentrations, 0.5 ml fill volume) for maintenance dose in Weeks 7–16. The placebo solution (isotonic sodium chloride) was filled into identical vials or pre-filled syringes. In accordance with the doses recommended by the prescribing information [1], up to 1.0 mg of semaglutide was administered for dose escalation in an open-label fashion via a single-patient-use pen that delivered 0.25 or 0.5 mg per injection in Weeks 1–9, and by a single-patient-use pen that delivered 1.0 mg per injection for maintenance dose in Weeks 10–16. Survodutide or placebo were administered by subcutaneous injection in the abdomen. During the maintenance period, injections were administered at the study centre or self-administered by patients. Semaglutide was administered subcutaneously in the abdomen, thigh or upper arm. Patients who discontinued the study during the treatment period were encouraged to complete an end of treatment visit, and a follow-up visit was scheduled for 5 weeks after the last dose of study drug.

Safety and tolerability were assessed based on the general occurrence of treatment-emergent adverse events, safety laboratory parameters, physical examination, vital sign measurements and 12-lead ECG. ECG services were externally provided and recordings were evaluated centrally.

Statistical analyses

The study aimed to demonstrate proof of clinical concept (PoCC) regarding a non-flat dose–response curve for survodutide, and definition of a suitable dose-escalation scheme and dose range for survodutide in terms of efficacy, safety and tolerability. A multiple comparison procedure with modelling (MCPMod) approach was used [2], allowing for simultaneous evaluation of different potential dose–response patterns, while protecting against the overall probability of a type I error (one-sided $\alpha = 2.5\%$). For the contrast test (one-sided $\alpha = 2.5\%$), a sample size of 45 evaluable patients per dose group (DG) was needed to reach 94% average power for an assumed effect size of 0.6%. Calculations for the PoCC step were performed using the Dose Finding R-package with 1000 repetitions [3]. To account for the repeated nature of the data and covariates in the model, mixed model for repeated measures (MMRM) analysis was carried out and covariate-adjusted, least square means estimates of average response for each DG and the covariance matrix were extracted from the fit and used for MCPMod analysis. For the twice weekly dosing schemes, total dose per week was considered for the MCPMod analysis with a restricted maximum likelihood based approach using MMRM. Overall, the analyses included the fixed, categorical effects of treatment at each visit and the fixed continuous effects of baseline at each visit, with visit treated as the repeated measure and an unstructured covariance structure used to model within-subject measurements. The relative bodyweight change from baseline was evaluated using an MMRM model and MCP-Mod analysis in the same way as for the primary endpoint, and descriptive statistics. Absolute bodyweight change from baseline and change from baseline in waist circumference were assessed using an MMRM analysis as for the primary endpoint analysis, and descriptive statistics. The proportion of participants achieving ≥5% and ≥10% bodyweight reduction from baseline were analysed using a logistic regression and descriptive statistics. Patient reported outcomes (TFEQ-R18V2, VAS and PGI-S) were analysed by

MMRM, as for the primary endpoint, and descriptive statistics. All safety endpoints, NASH-related scores and SMBG were analysed descriptively.

ESM – Results

Patient-reported outcome questionnaires

No clear treatment effect was seen for the mean ± SD change from baseline in TFEQ-R18V2 parameters, with only minor changes observed in adjusted mean MMRM estimates for absolute change from baseline in cognitive restraint, emotional eating and uncontrolled eating (ESM Table 2). The changes observed in survodutide- and semaglutide-treated groups were not significant compared with placebo, except for the mean ± SE change from baseline in emotional eating score at Week 17 in DG5 (−6.98% ± 3.15; p=0.0274). The proportion of patients recording 'Not at all' for difficulty to control eating behaviours (via PGI-S) tended to increase in all DGs at Week 17 compared with baseline, including placebo (ESM Table 3). Hunger VAS score tended to decrease with survodutide treatment and semaglutide, although these changes were mostly minor, and scores were highly variable with marked discrepancies between the means and medians in all treatment groups (ESM Table 2). Changes from baseline were observed in DGs 4 and 6 at Week 17, with a median change of −8.0 observed in both groups.

References

1. Semaglutide (Ozempic) Prescribing Information. Novo Nordisk A/S. 2021.
2. Pinheiro J, Bornkamp B, Bretz F (2006) Design and analysis of dose-finding studies combining multiple comparisons and modeling procedures. J Biopharm Stat 16(5):639-656. https://doi.org/10.1080/10543400600860428
3. Bornkamp B, Pinheiro J, Bretz F, Sandig L (2023) Planning and analyzing dose finding experiments. Available from https://cran.r-project.org/web/packages/DoseFinding/DoseFinding.pdf. Accessed 26 January 2023

Tables

ESM Table 1 Absolute change from baseline in TFEQ-R18V2 parameters and Hunger VAS score at end of treatment

	Survodutide 0.3 mg qw (*n*=50)	Survodutide 0.9 mg qw (*n*=50)	Survodutide 1.8 mg qw (*n*=52)	Survodutide 2.7 mg qw (*n*=50)	Survodutide 1.2 mg biw (*n*=51)	Survodutide 1.8 mg biw (*n*=49)	Semaglutide 1.0 mg qw (*n*=50)	Placebo (*n*=59)
TFEQ-R18V2, mean ± SD								
Cognitive restraint score	1.06 ± 25.24	2.13 ± 28.90	−1.71 ± 24.78	5.39 ± 30.19	0.99 ± 21.95	4.20 ± 25.84	3.14 ± 23.50	−2.27 ± 30.60
Emotional eating score	−6.75 ± 17.76	−3.07 ± 18.49	−8.26 ± 13.95	−12.12 ± 16.87	−14.20 ± 19.16	−7.21 ± 22.29	−9.57 ± 18.96	−5.67 ± 24.68
Uncontrolled eating score	−9.70 ± 15.80	−8.67 ± 18.67	−11.11 ± 14.22	−11.0 ± 15.31	−11.77 ± 13.13	−10.19 ± 17.49	−9.20 ± 14.36	−8.24 ± 21.40
Hunger VAS, mean ± SD	0.4 ± 19.9	−2.7 ± 26.3	−8.9 ± 31.1	−5.1 ± 30.5	−8.4 ± 25.3	−9.4 ± 32.4	−3.4 ± 24.6	−1.5 ± 29.5
Comparison vs placebo								
TFEQ-R18V2, adjusted mean ± SE								
Cognitive restraint score	−0.71 ± 4.89	0.29 ± 4.82	−0.10 ± 4.95	2.51 ± 5.18	1.83 ± 4.84	−1.20 ± 5.07	1.59 ± 4.83	–
Emotional eating score	−3.43 ± 3.19	5.21 ± 3.14	−4.32 ± 3.22	−6.54 ± 3.35	−6.98 ± 3.15*	−2.04 ± 3.28	−3.05 ± 3.14	–
Uncontrolled eating score	−1.72 ± 2.84	1.78 ± 2.80	−1.89 ± 2.88	−2.44 ± 3.00	−1.35 ± 2.81	−1.77 ± 2.95	−1.23 ± 2.80	–

biw, twice weekly; qw, once weekly; VAS, visual analogue scale.

*p=0.0274

ESM Table 2 Change from baseline in PGI-S for difficulty to control eating behaviours

		PGI-S				
		Not at all	Mild	Moderate	Severe	Very severe
Survodutide 0.3 mg qw (*n*=50)	Baseline	19 (38.0)	9 (18.0)	18 (36.0)	4 (8.0)	0
	EoT	19 (45.2)	11 (26.2)	10 (23.8)	2 (4.8)	0
Survodutide 0.9 mg qw (*n*=50)	Baseline	12 (24.0)	16 (32.0)	20 (40.0)	2 (4.0)	0
	EoT	20 (42.6)	12 (25.5)	15 (31.9)	0	0
Survodutide 1.8 mg qw (*n*=52)	Baseline	14 (26.9)	13 (25.0)	22 (42.3)	2 (3.8)	1 (1.9)
	EoT	21 (53.58)	9 (23.1)	9 (23.1)	0	0
Survodutide 2.7 mg qw (*n*=50)	Baseline	20 (40.0)	15 (30.0)	14 (28.0)	1 (2.0)	0
	EoT	20 (60.6)	9 (27.3)	3 (9.1)	1 (3.0)	0
Survodutide 1.2 mg biw (*n*=51)	Baseline	10 (19.6)	14 (27.5)	25 (49.0)	2 (3.9)	0
	EoT	22 (48.9)	15 (33.3)	8 (17.8)	0	0
Survodutide 1.8 mg biw (*n*=49)	Baseline	12 (24.5)	19 (38.8)	15 (30.6)	3 (6.1)	0
	EoT	19 (51.4)	15 (40.5)	3 (8.1)	0	0
Semaglutide 1.0 mg qw (*n*=50)	Baseline	20 (40.0)	10 (20.0)	17 (34.0)	3 (6.0)	0
	EoT	29 (63.0)	9 (19.6)	7 (15.2)	1 (2.2)	0
Placebo (*n*=59)	Baseline	17 (28.8)	11 (18.6)	29 (49.2)	2 (3.4)	0
	EoT	27 (55.1)	7 (14.3)	13 (26.5)	2 (4.1)	0

Data are presented as the frequency of patients reporting each category; *n* (%).

biw, twice weekly; EoT, end of treatment; PGI-S, Patient's Global Impression of Severity; qw, once weekly.

ESM Table 3 Summary of serious AEs

	Survodutide 0.3 mg qw (*n*=50)	Survodutide 0.9 mg qw (*n*=50)	Survodutide 1.8 mg qw (*n*=52)	Survodutide 2.7 mg qw (*n*=50)	Survodutide 1.2 mg biw (*n*=51)	Survodutide 1.8 mg biw (*n*=49)	Semaglutide 1.0 mg qw (*n*=50)	Placebo (*n*=59)	Total survodutide (*n*=302)
Total patients with serious AEs	1 (2.0)	4 (8.0)	3 (5.8)	2 (4.0)	1 (2.0)	0	0	3 (5.1)	11 (3.6)
Abdominal pain	1 (2.0)[a]	0	0	0	0	0	0	0	1 (0.3)
Diarrhoea	0	0	0	1 (2.0)[a]	0	0	0	0	1 (0.3)
Irritable bowel syndrome	0	1 (2.0)[b]	0	0	0	0	0	0	1 (0.3)
Mouth ulceration	0	1 (2.0)[a,b]	0	0	0	0	0	0	1 (0.3)
Vomiting	1 (2.0)[a]	0	0	0	0	0	0	0	1 (0.3)
Inguinal hernia	0	0	0	0	0	0	0	1 (1.7)	0
Cellulitis	0	2 (4.0)	0	0	0	0	0	0	2 (0.7)
Viraemia	0	0	0	1 (2.0)	0	0	0	0	1 (0.3)
IIIrd nerve paralysis	0	0	1 (1.9)	0	0	0	0	0	1 (0.3)
Paraparesis	0	0	1 (1.9)[b]	0	0	0	0	0	1 (0.3)
Autoimmune disorder	0	1 (2.0)[a,b]	0	0	0	0	0	0	1 (0.3)

Dehydration	0	0	1 (1.9)[a]	0	0	0	0	0	1 (0.3)
Arthralgia	0	0	0	0	1 (2.0)	0	0	0	1 (0.3)
Back pain	0	0	0	0	1 (2.0)	0	0	0	1 (0.3)
Pharyngeal ulceration	0	1 (2.0)[a,b]	0	0	0	0	0	0	1 (0.3)
Cholecystitis	0	0	0	0	0	0	0	1 (1.7)	0
Hypotension	0	0	0	0	0	0	0	1 (1.7)	0

Data are presented as *n* (%).

[a]Assessed by investigator to be drug-related. [b]AE did not resolve until the completion of the trial.

AE, adverse event; biw, twice weekly; qw, once weekly.

Figures

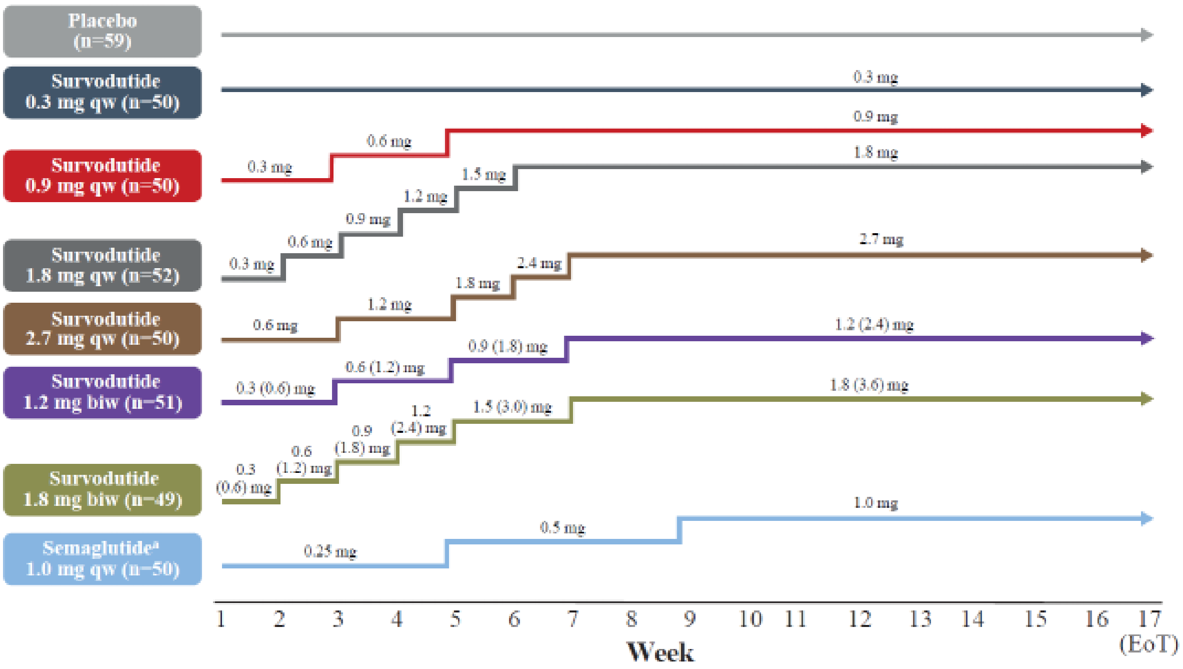

ESM Fig. 1 Dose-escalation schemes.

[a]Semaglutide arm is open label. Numbers in brackets signify total weekly dose.

biw, twice weekly; EoT, end of treatment; qw, once weekly.

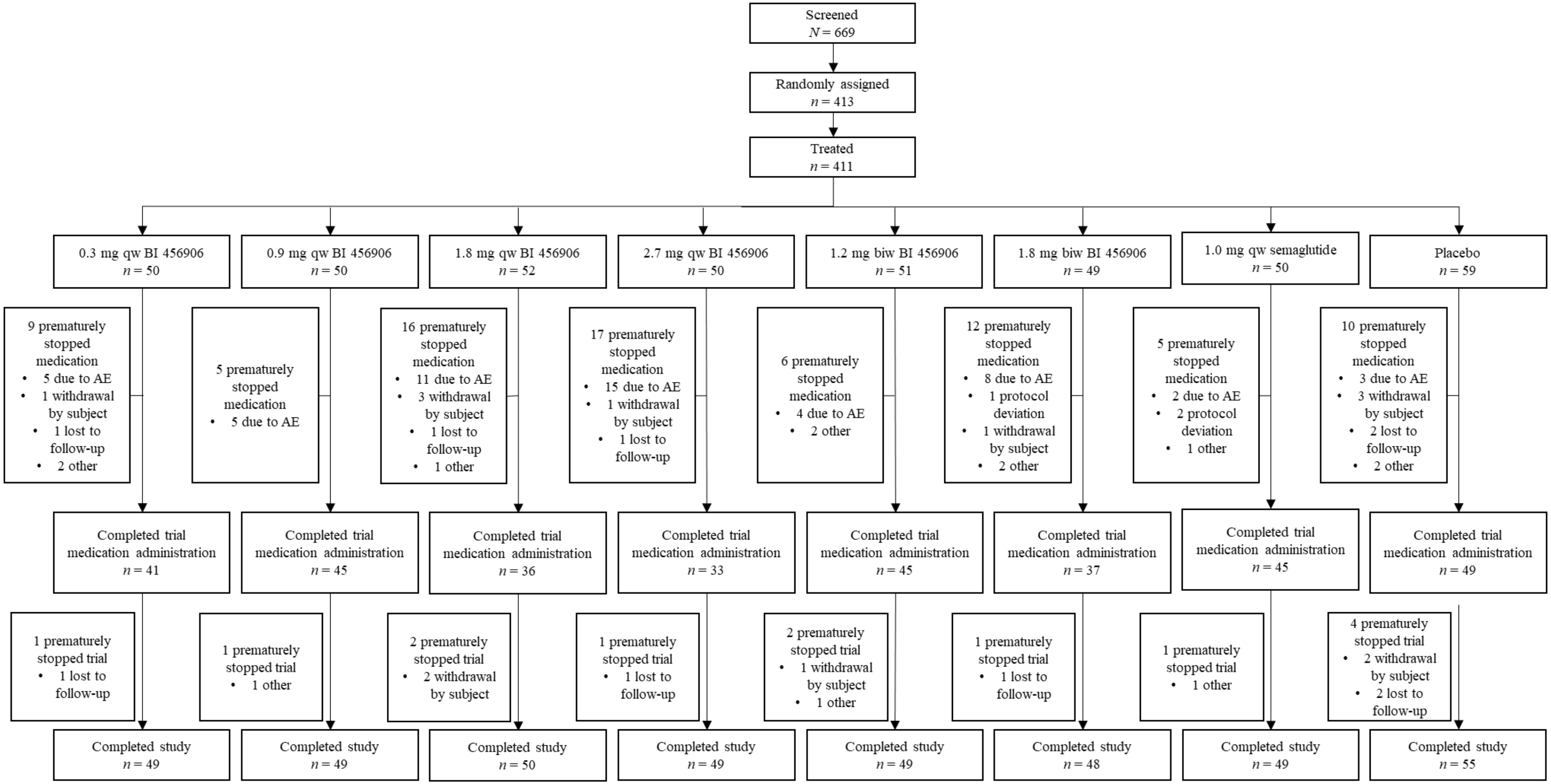

ESM Fig. 2 Subject disposition flow diagram.

AE, adverse event; biw, twice weekly; qw, once weekly.

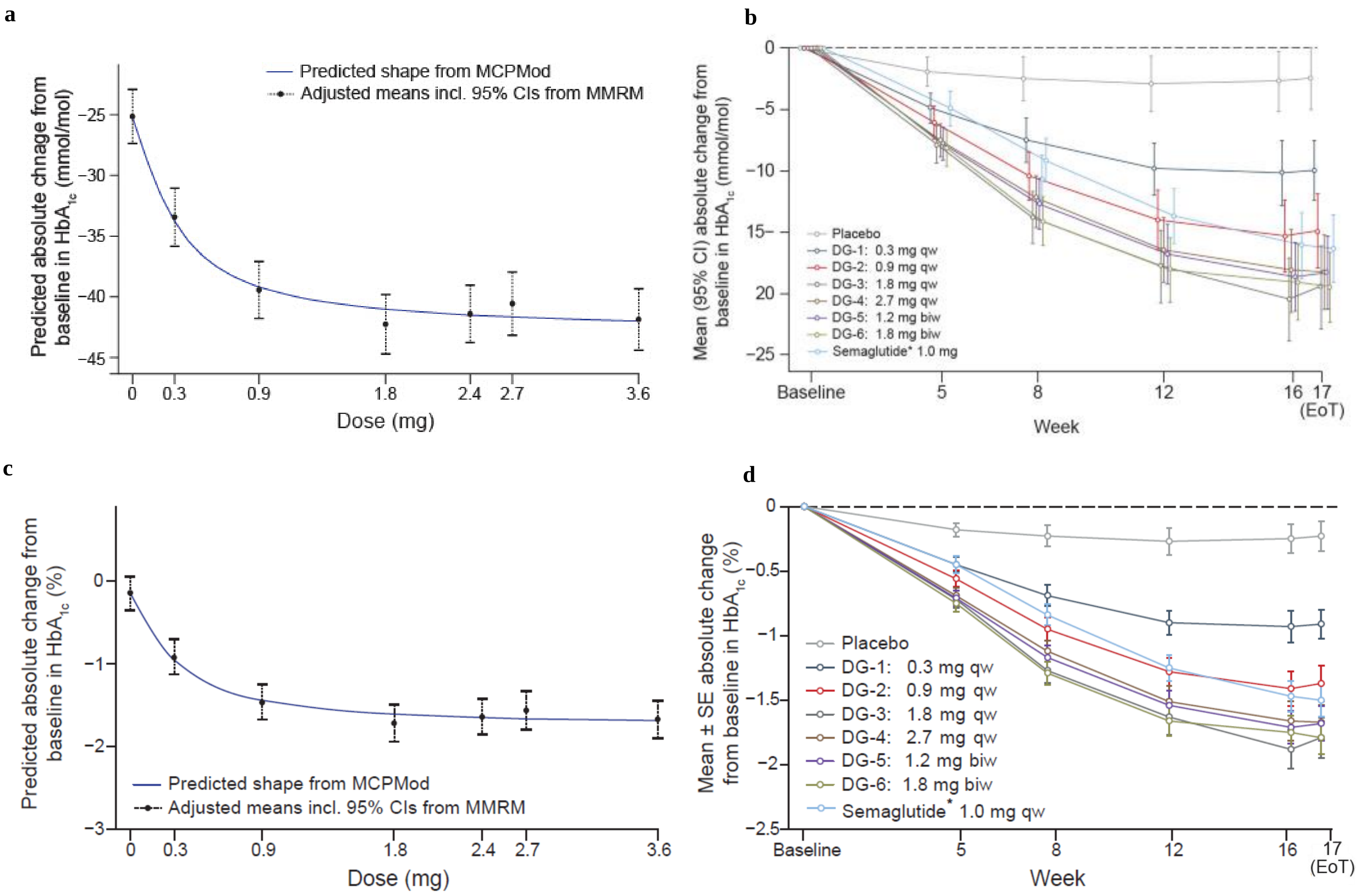
a
Predicted shape from MCPMod
Adjusted means incl. 95% CIs from MMRM
Predicted absolute chnage from baseline in HbA1c (mmol/mol)
−25
−30
−35
−40
−45
0
0.3
0.9
1.8
2.4
2.7
3.6
Dose (mg)
b
Mean (95% CI) absolute change from baseline in HbA1c (mmol/mol)
0
−5
−10
15
20
−25
Placebo
DG-1: 0.3 mg qw
DG-2: 0.9 mg qw
DG-3: 1.8 mg qw
DG-4: 2.7 mg qw
DG-5: 1.2 mg biw
DG-6: 1.8 mg biw
Semaglutide* 1.0 mg
Baseline
5
8
12
16
17
(EoT)
Week
c
Predicted absolute change from baseline in HbA1c (%)
0
−1
−2
−3
Predicted shape from MCPMod
Adjusted means incl. 95% CIs from MMRM
0
0.3
0.9
1.8
2.4
2.7
3.6
Dose (mg)
d
Mean ± SE absolute change from baseline in HbA1c (%)
0
−0.5
−1
−1.5
−2
−2.5
Placebo
DG-1: 0.3 mg qw
DG-2: 0.9 mg qw
DG-3: 1.8 mg qw
DG-4: 2.7 mg qw
DG-5: 1.2 mg biw
DG-6: 1.8 mg biw
Semaglutide* 1.0 mg qw
Baseline
5
8
12
16
17
(EoT)
Week

ESM Fig. 3 Change from baseline in HbA_{1c}. (**a**) Predicted dose response of the change from baseline in HbA_{1c} (mmol/mol) after 16 weeks' treatment with survodutide, MCPMod averaging model. (**b**) Descriptive statistics for the absolute change from baseline in HbA_{1c} (mmol/mol) over time. (**c**) Predicted dose response of the change from baseline in HbA_{1c} (%) after 16 weeks' treatment with survodutide, MCPMod averaging model. (**d**) Descriptive statistics for the absolute change from baseline in HbA_{1c} (%) over time.

[a]Semaglutide arm is open label.

biw, twice weekly; DG, dose group; EoT, end of treatment; MCPMod, multiple comparison procedure with modelling techniques; MMRM, mixed model repeated measures; qw, once weekly.

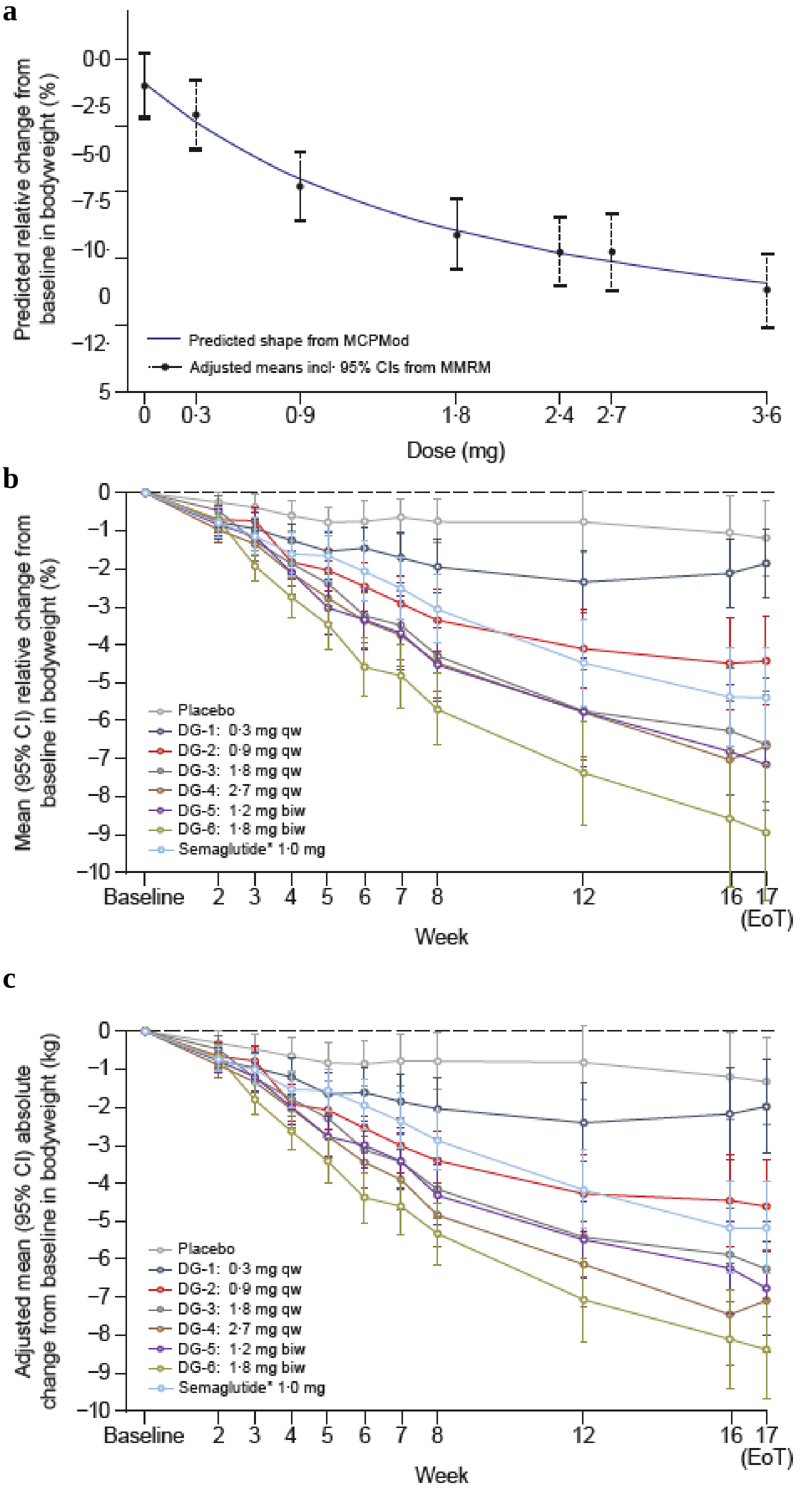

ESM Fig. 4 Change from baseline in bodyweight. (**a**) Predicted dose response of the relative change from baseline in bodyweight (%) after 16 weeks of treatment with survodutide, MCPMod averaging model. (**b**) Descriptive statistics for the relative change from baseline in

bodyweight (%) over time. (**c**) MMRM estimates for the absolute change from baseline in bodyweight (kg) over time.

[a]Semaglutide arm is open label.

biw, twice weekly; DG, dose group; EoT, end of treatment; MCPMod, multiple comparison procedure with modelling techniques; MMRM, mixed model repeated measures; qw, once weekly.

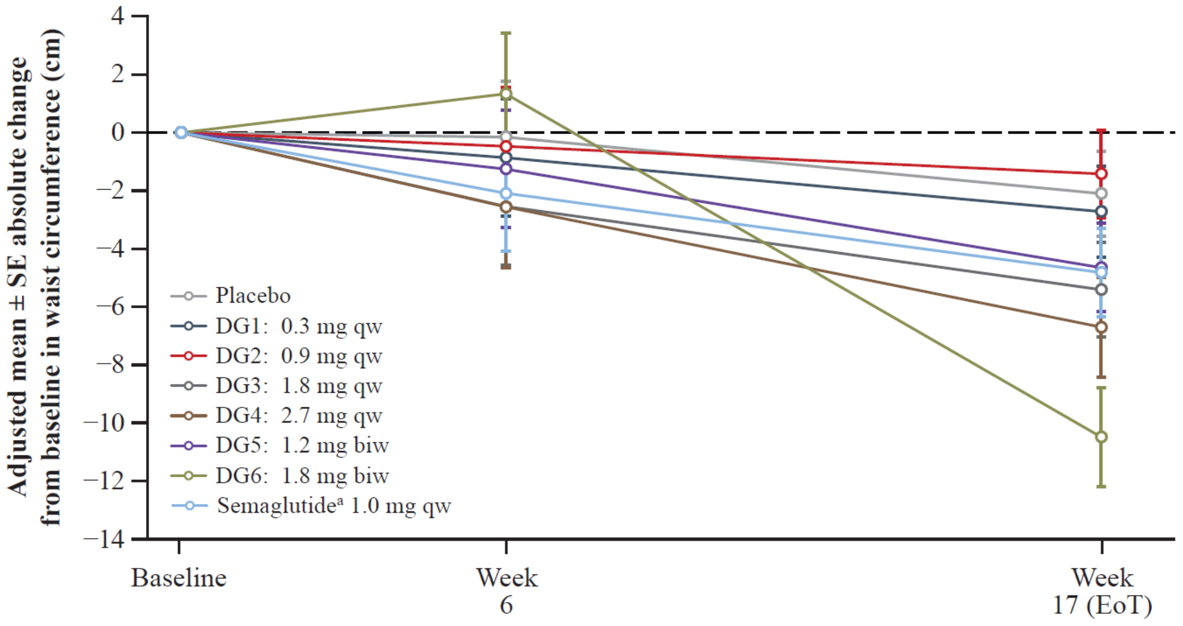

ESM Fig. 5 Absolute change in waist circumference from baseline.

[a]Semaglutide arm is open label.

biw, twice weekly; DG, dose group; EoT, end of treatment; qw, once weekly.

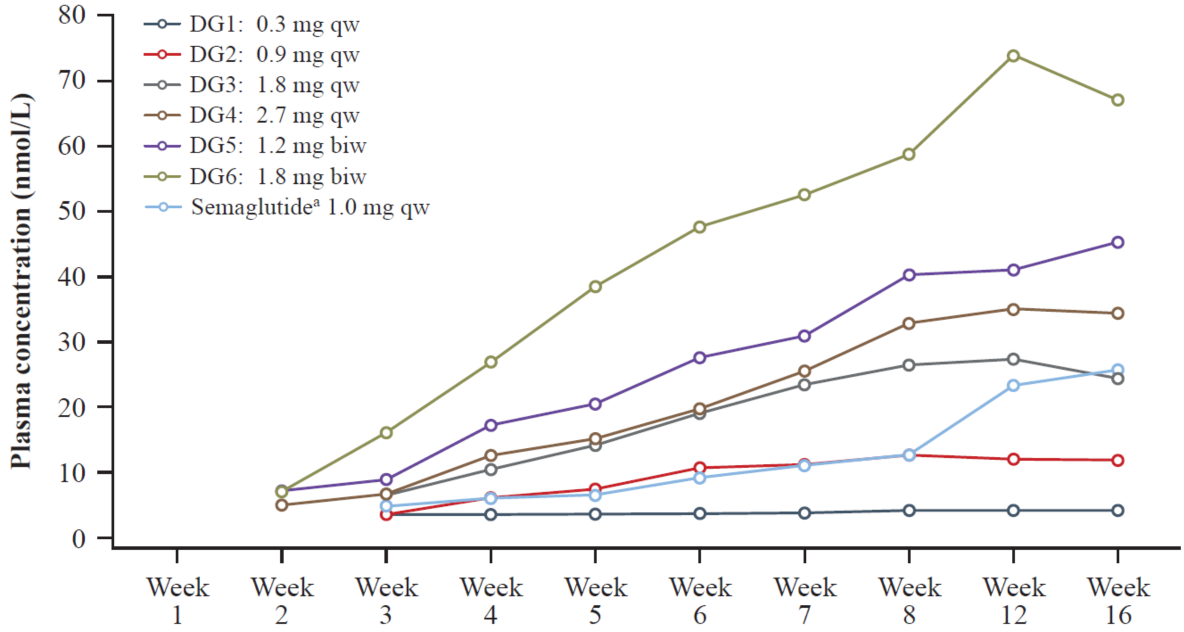

ESM Fig. 6 Geometric mean plasma concentration–time profiles of survodutide and semaglutide.

[a]Semaglutide arm is open label.

biw, twice weekly; DG, dose group; qw, once weekly.

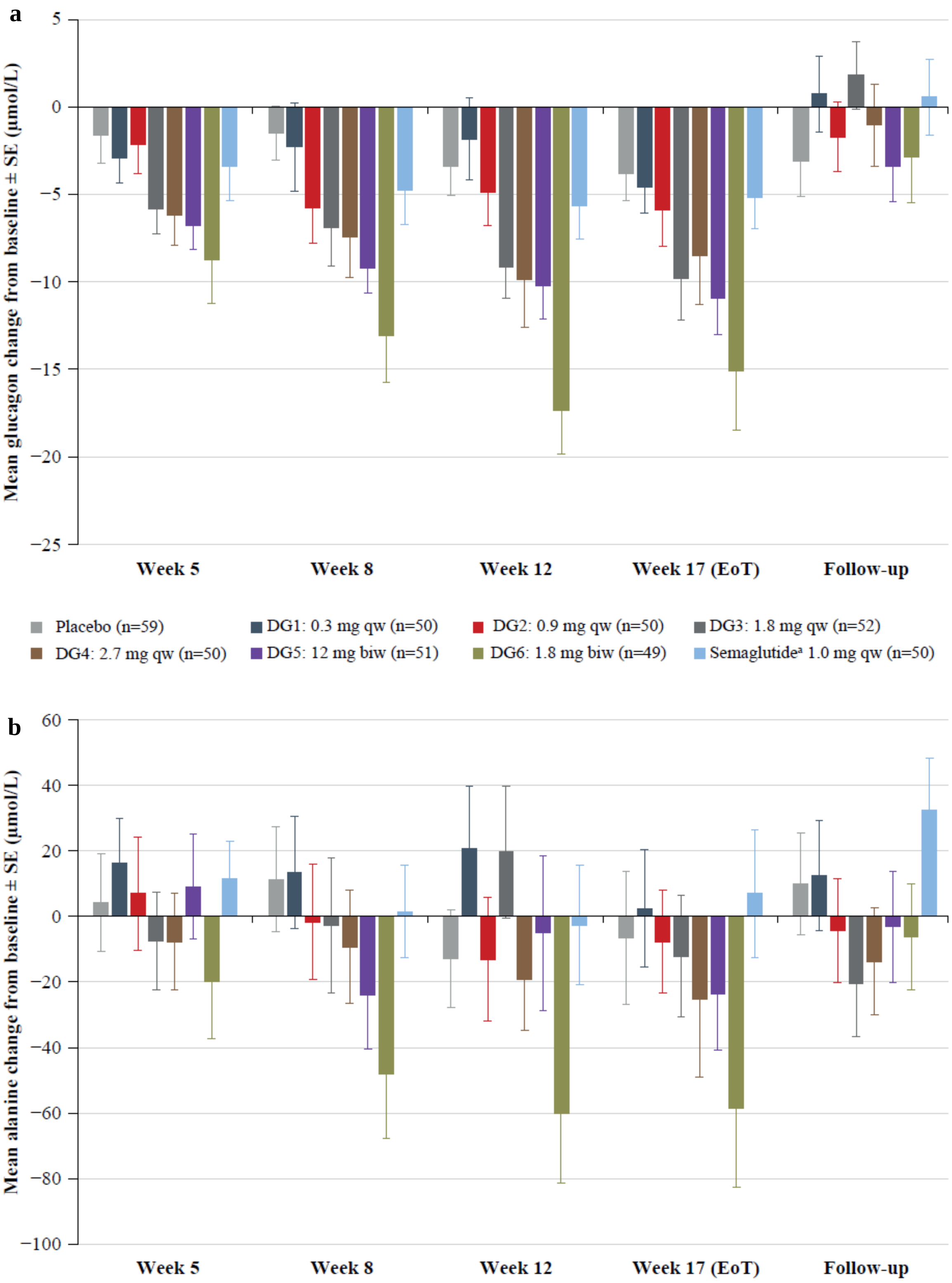

ESM Fig. 7 Mean absolute change from baseline in (**a**) plasma glucagon and (**b**) alanine.

[a]Semaglutide arm is open label.

biw, twice weekly; DG, dose group; EoT, end of treatment; qw, once weekly.

List of trial sites and investigators

Centre number	Investigator name and role	Address
**Australia**		
AUS1	Principal Investigator: Caterson Prof (BSc (Med)), Ian<br><br>Sub-Investigator(s): Hocking Dr., Samantha Lau Dr., Namson Markovic, Tania	Boden Institute of Obesity, Nutrition, Exercise and Eating Disorders Charles Perkins Center D 17 John Hopkins Drive Camperdown<br>New South Wales 2006<br>Australia
AUS2	Principal Investigator(s): Morbey Dr. (MBChB), Claire<br><br>Sub Investigator(s): Diez Alvarez Dr., Sergio	Hunter Diabetes Centre Level 1, 41 Llewellyn St Merewether<br>New South Wales 2291<br>Australia
AUS3	Principal Investigator(s): Cohen Dr. (MD), Neale<br><br>Sub Investigator(s): Sharifi Dr., Amin	Baker Heart and Diabetes Institute 99 Commercial Rd Melbourne Victoria 3004<br>Australia
AUS5	Principal Investigator(s): Shirazi Dr. (MD), Mitra<br><br>Sub Investigator(s): Montarello Dr., Nicholas Joseph	Royal Adelaide Hospital Clinical Trial Unit<br>Level 3<br>1 Port Road Adelaide<br>South Australia 5000<br>Australia
AUS8	Principal Investigator(s): Gilfillan Dr. (MBBS), Chris<br><br>Sub Investigator(s): Simpson Dr., Richard McCarthy Dr., Josephine Yee Dr., Ming Li Gordon, Debra	Monash University Monash University<br>Eastern Clinical Research Unit Level 2<br>5 Arnold Street Box Hill Victoria<br>3128<br>Australia
AUS7	Principal Investigator(s): Ekinci Dr. (MBBS), Elif<br><br>Sub-Investigator(s): Nash Dr., Ben<br>Neoh Dr., Sandra Seah Dr., Jas-mine Ali Dr., Aleena Price Dr., Sarah	Austin Health<br>Metabolic Disorders Service Heidelberg Repatriation Hospital<br>Level 1 Centaur Building, 198 Banksia St<br>Heidelberg Victoria 3081<br>Australia
**Austria**		
AUT1	Principal Investigator(s): Kautzky-Willer Prof. Dr. (MD), Alexandra<br><br>Sub-Investigator(s): Harreiter Dr., Juergen Leutner Dr., Michael	AKH - Medical University of Vienna<br>Universitätsklinik für Innere Medizin III<br>Abteilung für Endokrinologie und Stoffwechsel<br>Waehringer Guertel 18-20 Vienna<br>1090<br>Austria
AUT6	Principal Investigator(s): Ludvik Dr. (MD), Bernhard	KH Rudolfstiftung, 1. Med. Abt., Wien<br>Krankenanstalt Rudolfstiftung inkl. Semmelweis Frauenklinik 1.

	Sub-Investigator(s): Brix Dr., Johanna Maria Feder Dr., Astrid Hoellerl Dr., Florian Krzizek Dr., Eva-Christina Luger Dr., Philip Malek Dr., Sara Stoeckl, Alexander Gericke Dr., Greta Schrangl Dr., Christoph	Medizinische Abteilung<br>1. Medizinische Abteilung Juchgasse 25<br>Wien 1030<br>Austria
**Canada**		
CAN1	Principal Investigator(s): Akhras Dr. (MD), Ronald<br><br>Sub-Investigator(s): Daginawala Dr., Khatija	Centre Medical Acadie Suite 101A<br>9825 boul de l'Acadie Montreal<br>Quebec H4N 2W2<br>Canada
CAN3	Principal Investigator(s): Heaton Dr. (MD), Kenneth	Devonshire Clinical Research Inc. Devonshire Clinical Research Inc. Suite 301<br>423 Devonshire Avenue Woodstock<br>Ontario N4S 5P5<br>Canada
CAN4	Principal Investigator(s): Garg Dr. (MD), Naveen<br><br>Sub-Investigator(s): Breger Dr., Laurie Claveau Dr., Jessica	Manna Research (Montreal) Suite 145<br>175 Stillview Avenue Pointe-Claire<br>Quebec H9R 4S3<br>Canada
CAN5	Principal Investigator(s): Wharton Dr. (MD), Sean<br><br>Sub-Investigator(s): Greenspoon Dr., Allen Greenspoon Dr., Todd	The Wharton Medical Clinic Clinical Trials Inc. M14<br>414 Victoria Avenue North Hamilton<br>Ontario L8L 5G8<br>Canada
CAN6	Principal Investigator(s): Goldenberg Dr. (MD), Ronald<br><br>Sub-Investigator(s): D'Sylva Dr., Christopher Schlosser Dr., Robert Sionit Dr., David	LMC Clinical Research Inc. (Thornhill) Unit 5-10<br>1600 Steeles Avenue West Concord<br>Ontario L4K 4M2<br>Canada
CAN7	Principal Investigator(s): Bajaj Dr.(MD), Harpreet<br><br>Sub-Investigator(s): Aslam Dr., Nadeem Bahl Dr., Nupur Chandra Dr., Prakash Thobani Dr., Sanya	LMC Clinical Research Inc. (Brampton)<br>2979 Bovaird Drive East Brampton<br>Ontario L6S 0C6<br>Canada
CAN8	Principal Investigator(s): Vallieres Dr. (MD), Gerald<br><br>Sub-Investigator(s): Bissonnette Dr., Alain Blouin Dr., Francois	Manna Research (Quebec) Local 203<br>1190 A, Rue de Courchevel Levis<br>Quebec G6W 0M5<br>Canada
CAN9	Principal Investigator(s): Jones Dr. (MB, ChB), Michael<br><br>Sub-Investigator(s): Troughton Dr., Timothy	Cook Street Medical Clinic CookMed Research Suite 107 Suite 107<br><br>1175 Cook Street Victoria<br>British Columbia V8V 4A1<br>Canada

**Czech Republic**		
CZE2	Principal Investigator(s):<br>Vesela Dr. (MD), Alica<br><br>Sub-Investigator(s):<br>Krupicka MUDr., Tomas<br>Novakova MUDr., Edita<br>Vesely Dr., Jiri	Edumed s.r.o Smetanova 91 Broumov 55001<br>Czech Republic
CZE3	Principal Investigator(s):<br>Svacina Dr. (MD), Stepan<br><br>Sub-Investigator(s):<br>Matoulek Dr., Martin<br>Mikes Dr., Ondrej Zurkova Dr., Pavla	General Faculty Hospital, Prague U Nemocnice 2<br>Prague 2<br>128 08<br>Czech Republic
**Germany**		
DEU1	Principal Investigator(s):<br>Rose Dr. (MD), Ludger<br><br>Sub-Investigator(s):<br>Pohlmeier Dr., Harald	Institut für Diabetesforschung Münster GmbH<br>Institut für Diabetesforschung Hohenzollernring 70<br>Münster 48145<br>Germany
DEU3	Principal Investigator(s):<br>Schürholz Dr. (MD), Thomas	InnoDiab Forschung GmbH Eleonorastrasse 42<br>Essen 45136<br>Germany
DEU7	Principal Investigator(s):<br>Klausmann Dr. (MD), Gerhard	Studienzentrum Aschaffenburg Studienzentrum<br>Elisenstraße 26 Aschaffenburg 63739<br>Germany
**Hungary**		
HUN1	Principal Investigator Pall Dr. (MD), Denes<br><br>Sub-Investigator(s):<br>Kobling Dr., Tamas<br>Szentimrei Dr., Reka<br>Varadi Dr., Zita Zsiros, Noemi	University Debrecen Hospital Internal Medicine Clinic Nagyerdei krt. 98,<br>Debrecen 4032<br>Hungary
HUN2	Principal Investigator(s):<br>Koranyi Dr. (MD)., Laszlo<br><br>Sub Investigator(s): Schmid Dr., Anita Bezzegh Dr., Katalin Pauer Dr., Jozsef	DRC Gyogyszervizsgalo Kozpont Kft., Balatonfured<br>DRC Drug Research Center LLC Ady Endre street 12/B Balatonfured<br>8230<br>Hungary
HUN3	Principal Investigator(s):<br>Hermanyi Dr. (MD), Zsolt<br><br>Sub Investigator(s): Nadas Dr., Judit Vida Dr., Klara	Bajcsy-Zsilinszky Hospital and Clinic<br>IV. Department of Internal Medicine and Cardiology<br>Maglodi str. 89-91 Budapest<br>1106<br>Hungary
**Korea, Republic of**		
KOR1	Principal Investigator(s):<br>Kim Dr. (MD), Kyoung-Ah<br><br>Sub Investigator(s): Choi, Han Seok<br>Lee Dr., Seung Eun	Dongguk University Ilsan Hospital 27 Dongguk-ro, Ilsandong-gu Goyang<br>10326<br>Korea, Republic of
KOR2	Principal Investigator(s):<br>Kim Dr. (MD), Doo-Man	Kangdong Sacred Heart Hospital Hallym University Medical Center, Dept of Cardiology

	Sub Investigator(s): Kim Dr., Min Kyung	Hallym University Medical Center, Dept of Cardiology<br>445 Gildong, Kangdong-Ku Seoul<br>134701<br>Korea, Republic of
KOR3	Principal Investigator(s): Yoo Dr. (MD), Soon-Jib<br><br>Sub Investigator(s): Jeong, Heekyoung Lee, Seong-Su Son Dr., Jang Won	The Catholic University of Korea, Bucheon St.Mary's Hospital<br>The Catholic University of Korea, Bucheon St.Mary's Hospital<br>327-2, Sosa-ro, Wonmi-gu Bucheon<br>14647<br>Korea, Republic of
**New Zealand**		
NZL1	Principal Investigator(s): Titchener Dr. (MD), Janet<br><br>Sub Investigator(s): Lionnet Dr., Claudette Montgomery Dr., Barnaby Murphy Dr., Michael	Optimal Clinical Trials Level 2, 97 Grafton Rd Auckland<br>1010<br>New Zealand
NZL2	Principal Investigator(s): Quinn Dr. (MBChB), Dean<br><br>Sub Investigator(s): Joseph Dr., Joanna Stubbs Dr., Richard	P3 Research First Floor 121 Adelaide Rd Newtown Wellington NZ 6021<br>New Zealand
NZL3	Principal Investigator(s): Kamerbeek Dr. (MBChB), Jackie<br><br>Sub Investigator(s): Allen Dr., Katrina	P3 Research Promed house Suite 11<br>71 Tenth Ave Tauranga 3110<br>New Zealand
NZL4	Principal Investigator(s): Stubbs Dr. (MD), Richard<br><br>Sub Investigator(s): Joseph Dr., Joanna Pointing Dr., Nicola Poracchia Dr., Anne- Christine	P3 Research Kapiti 44A Ihakara Street Paraparaumu<br>5032<br>New Zealand
**Poland**		
POL1	Principal Investigator Strozik-Krecichwost Dr. (MD), Agnieszka<br><br>Sub-Investigator(s): Ramos Dr., Przemyslaw Kedzia Dr., Agnieszka	Vita Longa Sp. z o.o. Uniczowska 6 Katowice<br>40-748<br>Poland
POL10	Principal Investigator Klodawska Dr.(MD), Katarzyna<br><br>Sub-Investigator(s): Abu Yasin Dr., Najwa Kaczmarczyk Dr., Aneta Witas Dr., Beata	NBR Polska<br>ul. 29 Listopada 18A lok. 4 Warsaw<br>00-465<br>Poland
POL3	Principal Investigator Gorska Dr. (MD), Izabela<br><br>Sub-Investigator(s): Beben Dr., Anna	Clinical Research Centre Clinsante SC Ewa Galczak-Nowak private practice Galczynskiego 45<br>Torun 87-100<br>Poland

POL4	Principal Investigator Kujawska-Luczak Dr. (MD), Magdalena<br><br>Sub-Investigator(s): Cieslak Mr., Piotr Durka Mr., Emil Kwiatek Dr., Joanna Kwiatek Dr., Michał Pieronkiewicz Dr., Justyna Pruchniewski Dr., Lukasz	Pratia SA<br>Examen Sp. Z o.o. private practice Poznanska str. 14 Skorzewo<br>60-185<br>Poland
POL7	Principal Investigator Bronisz Dr. (MD), Agata<br><br>Sub-Investigator(s): Korbal-Piasecka Dr., Dominika<br>Piasecki Dr., Piotr Wasilow Dr., Karolina Wieczor, Radosław<br>Zmudzinska Dr., Malgorzata	In-Vivo Sp. Z o.o. ul. Kaszubska 17H Bydgoszcz<br>85-048<br>Poland
**Spain**		
ESP1	Principal Investigator Tinahones Madueno Dr. (MD), Francisco Jose<br><br>Sub-Investigator(s): Martínez Alfaro Dra., Baldomera<br>Sebastian Ochoa Dra., Nerea	Hospital Virgen de la Victoria<br>Servicio de Endocrinología y Nutrición Campus de Teatinos S/N<br>29010, Málaga, España
ESP2	Principal Investigator Ampudia-Blasco Dr. (MD), Francisco Javier<br><br>Sub-Investigator(s): Vicente Casanova Dr., Antonio	Hospital Clínico Universitario de Valencia<br>Medicina Interna<br>Unidad de HTA y riesgo cardiovascular C/ Menendez Pelayo, 4<br>Valencia 46010<br>Spain
ESP3	Principal Investigator Soto González Dr. (MD), Alfonso<br><br>Sub-Investigator(s): Díaz Dra., Carmen Novoa Dra., Iria	Hospital A Coruña COMPLEJO HOSPITALARIO UNIVERSITARIO A CORUÑA (CHUAC)<br>Consultas Externas Endocrino, 3ª planta Xubias de Arriba, 84<br>A Coruña 15006<br>Spain
ESP4	Principal Investigator Brotons Dr. (MD), Carlos<br><br>Sub-Investigator(s): Fernández Dra., Diana	C.A.P. Sardenya<br>Centro de atención primaria C/ Sardenya, 466<br>Barcelona 08025<br>Spain
**Taiwan**		
TWN1	Principal Investigator(s): Huang Dr. (MD), Chien-Ning<br><br>Sub Investigator(s): Kornelius Dr., Edy Lo Dr., Shih Chang Yang Dr., Yi Sun	Chung Shan Medical University Hospital<br>No. 110, Sec. 1, Jianguo N. Rd., South District, Taichung Migration Data 40201<br>Taiwan
TWN3	Principal Investigator(s):	Kaohsiung Chang Gung Memorial Hospital

	Chen Dr. (MD) , Jung-Fu<br><br>Sub Investigator(s): Chang Dr., Chih Min Chen Dr., Chao Tung Chen Dr., Yung-Nien Huang Dr., WC Tsai Dr., Chia Jen Tsao Dr., Cheng Feng Yang Dr., Yi Ting Kuo Dr., Ming Chun Shen Dr., Feng-Chih	N0.123, Dapi Road, Niao-Sung Dist Kaohsiung<br>83301<br>Taiwan
TWN4	Principal Investigator Tu Dr. (MD), Shih-Te<br><br>Sub Investigator(s): Cheng Dr., Yu Fang Hsia Dr., Chien Hsun Hsu Dr., Shang Ren Kuo Dr., Jeng Fu<br>Lin Dr., Shi Dou Lin Dr., Szu Han Su Dr., Shih Li Wang Dr., Shu Yi Yu Dr., I Ruie	Chang-Hua Christian Hospital 135, Nanxiao Street, Changhua<br>500<br>Taiwan
**United Kingdom**		
GBR3	Principal Investigator(s): Price Dr., Hermione<br><br>Sub Investigator(s): Brewster Dr., Sarah Glass Dr., Ian Whicher Dr., Clare Colley Dr., Jack	Moorgreen Hospital Tom Rudd Unit<br>Southern Health NHS Foundation Trust Botley Road, West End<br>Southampton SO30 3JB<br>United Kingdom
GBR4	Principal Investigator(s): Capehorn Dr , Matthew S<br><br>Sub-Investigator(s): Byrne Dr., James Byrne Dr., John	Clifton Medical Centre, Rotherham The Health Village<br>Doncaster Gate Rotherham<br>S65 1DA<br>United Kingdom
GBR5	Principal Investigator(s): Butler Dr. (MBChB MRCGP), Mike<br><br>Sub-Investigator(s): Thompson Dr., Steven<br>Calvert Dr., John	Waterloo Medical Centre 178 Waterloo Road<br>Blackpool<br>FY4 3AD<br>United Kingdom
GBR6	Principal Investigator(s): Turner Dr. (MBChB), Wayne<br><br>Sub-Investigator(s): Thomason Dr., Philip	Burbage Surgery Tilton Road Burbage, Hinkley LE10 2SE<br>United Kingdom<br><br>Phone: 01455 634879<br>Fax: 01455 891264<br>Email: wayne.turner(at)burbagesurgery.co.uk
GBR8	Principal Investigator(s): Glover Dr., Victoria<br>Cartwright Dr. (MBBS MRCGP DRCOG DCH), Simon<br><br>Sub Investigator(s): Cartwright Dr. (MBBS MRCGP DRCOG DCH), Simon<br>Law Dr., Alison	White Horse Medical Practice Faringdon Medical Centre Volunteer Way<br>Faringdon SN7 7YU<br>United Kingdom
**United States**		

USA10	Principal Investigator<br>Cannon Dr.(MD), Kevin Dougherty<br><br>Sub-Investigator(s):<br>Chadwick Dr.(MD), Mesha McKinney	PMG Research of Wilmington, LLC 1907 Tradd Court<br>Wilmington North Carolina 28401<br>United States
USA11	Principal Investigator<br>Oliver Dr.(DO), Michael J.<br><br>Sub-Investigator(s):<br>Everhart Dr.(MD), Brian G.<br>Jackson Dr.(MD), Timothy L.<br>Rust Dr.(MD), William J.<br>Sniezek Dr.(DO), Matthew P.	Heritage Valley Medical Group, Inc. 500 Sharon Road<br>Beaver Pennsylvania 15009<br>United States
USA12	Principal Investigator<br>Smith Dr.(MD), Timothy Robert<br><br>Sub-Investigator(s):<br>Maryas, Elizabeth M.<br>Rocco, Sheri L. Stoneman, Jill L. Straatmann, Donna M.	StudyMetrix Research, LLC 3862 Mexico Road<br>St. Peters Missouri 63303<br>United States
USA13	Principal Investigator<br>Lefebvre Dr.(MD), Gigi Claire<br><br>Sub-Investigator(s):<br>Burke Dr.(MD), Deborah A. O'Connell, Margaret Elizabeth<br>Wick, Natasha Nicole<br>Ahrens, Tracy Ferrante, John R.	Meridien Research 4751 66th Street North Saint Petersburg Florida<br>33709<br>United States
USA14	Principal Investigator<br>Taylor Dr.(MD), Kelly N.<br><br>Sub-Investigator(s):<br>Bowman Dr.(MD), William Kelly	Sensible Healthcare, LLC 2700 Old Winter Garden Road Ocoee<br>Florida 34761<br>United States
USA16	Principal Investigator<br>Ard Dr.(MD), Jamy Darone<br><br>Sub-Investigator(s): Darapu Dr.(MD), Hima<br>Jayaprakash Dr.(MD), Marionette	Wake Forest University Health Sciences<br>Wake Forest Baptist Medical Center 1 Medical Center Boulevard Winston-Salem<br>North Carolina 27157<br>United States
USA17	Principal Investigator<br>Crandall (FNP), Lisa Marie<br>Shah Dr.(MD), Suresh Hirji<br><br>Sub-Investigator(s):<br>Crandall (FNP), Lisa Marie<br>Patel Dr.(MD), Shashank C. Mughal Dr.(MD), Gul R. Richardson (RN), Kellie S. Shah Dr.(MD), Ravi S.	In-Quest Medical Research, LLC Suite 100<br>3135 Mathis Airport Parkway Suwanee<br>Georgia 30024<br>United States

USA18	Principal Investigator<br>Wilson Dr.(DO), Jonathan P.<br><br>Sub-Investigator(s):<br>Bennett, Gerri<br>Holmes Dr.(MD), Robert John<br>Smith Dr.(MD), Brian Stanley<br>Reynolds, Danielle F.<br>Smith, Holly Campbell	PMG Research of Winston-Salem, LLC Suite 306 1901 South Hawthorne Road Winston-Salem<br>North Carolina 27103<br>United States
USA19	Principal Investigator<br>Machineni Dr.(MD), Sriram<br><br>Sub-Investigator(s):<br>Buse Dr.(MD, PhD), John Bernard<br>Diner, Jamie Crystal<br>Dostou Dr.(MD), Jean Marie Fraser, Rachael<br>Kass, Alexander M.<br>Young Dr.(MD, PhD), Laura Anne<br>Bergamo, Katherine A<br>Dezube, Milana<br>Kirkman Dr.(MD), Marian Sue<br>Purrington, Ginna<br>Sweeney, Megan Uehling, Julie	The University of North Carolina at Chapel Hill UNC Diabetes Care Center Clinical Trials Unit 100 Eastowne Drive Chapel Hill<br>North Carolina 27514<br>United States
USA2	Principal Investigator<br>Manning Dr.(MD), Rickey D.<br><br>Sub-Investigator(s): Bell, Lori<br>Hurst Dr.(MD), James Wilson<br>Maupin, Ivey<br>Sturgeon Dr.(MD), Rodney E.<br>Adams, Kristi Broome, Stephanie	PMG Research of Knoxville PMG Research, Inc., d/b/a 4125 E. Emory Road Knoxville<br>Tennessee 37938<br>United States
USA22	Principal Investigator<br>Earl Dr.(MD), John Keith<br><br>Sub-Investigator(s):<br>Everhart, Nicole<br>Hart Dr.(MD), Robert Eric<br>Polster Dr., Scott David<br>Warta, Heather<br>Parker, Ryan Sherrill, Kristen	PMG Research of Hickory, LLC Suite 201<br>221 13th Avenue Place NW Hickory<br>North Carolina 28602<br>United States
USA23	Principal Investigator<br>Moran Dr.(MD), Joseph Edward<br><br>Sub-Investigator(s): Allred,	PMG Research of Piedmont Healthcare PMG Research, Inc. d/b/a PMG Research of Piedmont HealthCare

	Susan F. Danner, Stephanie Georgeson, Christy Rector Hatharasinghe Dr.(MD), Roger Amal Roshan Lail, Cortney Frankhouser Dr.(DO), Tara Lynn	138 Sherlock Drive Statesville North Carolina 28625 United States
USA25	Principal Investigator Pucillo Dr.(MD), Ronald<br><br>Sub-Investigator(s): Miller, Tracey Penson, Stephanie	Javara Research Suite 40 1111 Highway 6 Sugar Land Texas 77478 United States
USA28	Principal Investigator Frias Dr.(MD), Juan Pablo<br><br>Sub-Investigator(s): Abaniel, Rea Elizabeth Cruz Grio Dr.(MD), Maricor C Jenders Dr.(MD), Robert Allen Leibowitz Dr.(MD), Mark Todd Nelson (PA-C), John Syal Dr.(MD), Parvin D. Kelly, Sharon Kay	National Research Institute Suite 302 2010 Wilshire Boulevard Los Angeles California 90057 United States
USA29	Principal Investigator Bhargava Dr.(MD), Anuj<br><br>Sub-Investigator(s): Burke (PA), Christine Fitzgerald, Kathleen Wright, Diana	Iowa Diabetes and Endocrinology Research Center Suite 2 1031 Office Park Road West Des Moines Iowa 50265 United States
USA31	Principal Investigator Denham Dr.(DO, CPI), Douglas Scott<br><br>Sub-Investigator(s): Avworo, Ayoade Hedges Dr., Parke Joseph Cruz, Jennifer Korte, Matthew Nakazwe, Muziya	Clinical Trials of Texas, LLC Suite 200 5430 Fredericksburg Road San Antonio Texas 78229 United States
USA32	Principal Investigator Rosenstock Dr.(MD), Julio<br><br>Sub-Investigator(s): Banarer Dr.(MD), Salomon Boyles (MSN, APRN-CNP), Lauren M. Kniffen, Wendy Mora Dr. (MD), Pablo F.	Dallas Diabetes and Endocrine Center Dallas Diabetes Research Center C-685 7777 Forest Lane Dallas Texas 75230 United States
USA34	Principal Investigator Hsia Dr.(MD), Stanley Hsin- Wei<br><br>Sub-Investigator(s): Frias Dr.(MD), Juan Pablo Jenders Dr.(MD), Robert Allen Leibowitz Dr.(MD), Mark Todd	National Research Institute Suite 116 6011 Pacific Boulevard Huntington Park California 90255 United States

	Leon, Tricia	
USA38	Principal Investigator<br>Pullman Dr.(MD), John<br><br>Sub-Investigator(s):<br>Chamberlain Dr.(MD), David P.<br>Williams-Leber, Erin	Mercury Street Medical Group, PLLC 300 West Mercury Street<br>Butte Montana 59701<br>United States
USA39	Principal Investigator<br>Butuk Dr.(MD), David J.<br><br>Sub-Investigator(s):<br>Barney, Janelle Brennan, Erin Conrad, Melissa Frisby, Scott Mangum, Meredith<br>McHugh Dr.(MD), Timothy E.<br>Haft, Annelise Stephenson, Mandi	Solaris Clinical Research Suite 100<br>1525 East Leighfield Drive Meridian<br>Idaho 83646<br>United States
USA4	Principal Investigator<br>Nardandrea Dr.(MD), John Paul<br><br>Sub-Investigator(s):<br>Clevinger Dr.(MD), Sidney Ernest<br>Cone, Sharon Lynn Craggs, Lynn M. Griffis, Jordan Marie Henderson, Debra A. Hughes, Amy M. Klein, Jay Koppenhafer, Rebecca Suzanne	Renstar Medical Research 21 NE 1st Avenue<br>Ocala Florida 34470<br>United States
USA40	Principal Investigator<br>Cortes-Maisonet Dr.(MD), Gregorio A.<br><br>Sub-Investigator(s):<br>Allende-Vigo Dr.(MD), Myriam Z.<br>Vila-Rivera Dr.(MD), Karina	GCM Medical Group, PSC<br>62 Calle Jose Marti, Urb. Floral Park San Juan<br>Puerto Rico 00917<br>United States
USA41	Principal Investigator<br>Rubino Dr.(MD), John<br><br>Sub-Investigator(s): Harris Dr.(MD), Tina Griffin<br>Jacobs Dr.(MD), William Richard<br>Short Dr.(MD), Douglas Brian Ellis<br>Siddiqui Dr.(MD), Adeel Mohammad<br>Stoneback, Stephanie<br>Utech, Sarah Edmundson Dr.(MD), Warner Lee<br>Wells Galpin, Kelcie	PMG Research of Raleigh, LLC Suite 100<br>3521 Haworth Drive Raleigh<br>North Carolina 27609<br>United States
USA42	Principal Investigator:<br>Michael John Lillestol, MD	Lillestol Research, LLC Suite 101<br>4450 31st Avenue S Fargo<br>North Dakota 58104

	Sub-Investigators: Jamie M. Brown Karissa M. Emerson	United States
USA45	Principal Investigator Cardona Dr.(MD), Jose Francisco<br><br>Sub-Investigator(s): Castro Someillan (APRN), Yisel<br>Sosa Dr.(MD), Evelio H.	Indago Research and Health Center, Inc.<br>Suite 300<br>3700 West 12 Avenue Hialeah<br>Florida 33012<br>United States
USA47	Principal Investigator Momoyama Dr.(MD), Yoko Jabbar Dr.(MD), Zaid W.<br><br>Sub-Investigator(s): Fachet (RN), Karen Hughes, Emma Wright Dr.(MD), LaBianca Dinh (APN), Janie<br>Doyle (APN), Kimberly Ferrufino, Natalie Kruchten, Kenneth Murtaugh (RN), Joan Pua Dr.(MD), Jeffrey	DuPage Medical Group, Ltd Suite 130<br>1801 South Highland Avenue Lombard<br>Illinois 60148<br>United States
USA48	Principal Investigator Andersen Dr.(MD), James L.<br><br>Sub-Investigator(s): Fearon, Szheckera Figueroa, Colleen Francisco, Mae Ann Holmes, Diana Medina, Amy<br>Negron-Munoz Dr.(MD), Rosa E.<br>Gerber, Candice	Meridien Research<br>2135 Harden Boulevard Lakeland<br>Florida 33803<br>United States
USA49	Principal Investigator Acosta Dr.(MD), Idalia Ana<br><br>Sub-Investigator(s): Cuesta Torres Dr.(MD), Maria Romero Basso Dr.(MD), Sandor Andres	San Marcus Research Clinic, Inc. 16420 NW 59th Avenue<br>Miami Florida 33014<br>United States
USA5	Principal Investigator McCartney Dr.(MD), Michael J.<br><br>Sub-Investigator(s): Kennedy, Ashley<br>Rizos Dr.(DO), Demetrius P. Rosario, Alfaurys Sampson, MaryBeth M. Sharma, Mahima<br>Shay Dr.(MD), Marc	ActivMed Practices & Research, LLC Suite 203<br>421 Merimack Street Methuen Massachusetts 01844<br>United States
USA50	Principal Investigator Lucas Dr.(MD), Kathryn Jean	Lucas Research, Inc. 623 North 35th Street Morehead City<br>North Carolina 28557

	Sub-Investigator(s): Bell (FNP), Elizabeth Virginia Buery (PA-C), Andrea Lauren Smith (PA-C), Justin Norwood Hainsworth Dr.(DO), Jeffrey Theodore	United States
USA51	Principal Investigator Azizad Dr.(MD), Masoud M.<br><br>Sub-Investigator(s): Chow Dr.(MD), Christopher G. Nakatani (FNP-C), Lisa M. Smallson (FNP-BC), Monique	Valley Clinical Trials, Inc.<br>#203, #208, #210<br>18433 Roscoe Boulevard Northridge<br>California 91325<br>United States
USA9	Principal Investigator Nakhle Dr.(MD), Samer<br><br>Sub-Investigator(s): Monrreal, Amy Neylon, Ellen Palal Dr.(MD), Betsy Rosario, Adriana Ruiz	Palm Research Center, Inc. Suite 306<br>9280 West Sunset Road Las Vegas<br>Nevada 89148<br>United States
